# Utility of Quantitative EEG in Neurological Emergencies and ICU Clinical Practice

**DOI:** 10.3390/brainsci14090939

**Published:** 2024-09-20

**Authors:** Misericordia Veciana de las Heras, Jacint Sala-Padro, Jordi Pedro-Perez, Beliu García-Parra, Guillermo Hernández-Pérez, Merce Falip

**Affiliations:** 1 Neurology Service, Neurophysiology Department, Hospital Universitari de Bellvitge-IDIBELL, Universitat de Barcelona, 08908 L’Hospitalet de Llobregat, Barcelona, Spain; jpedro@bellvitgehospital.cat (J.P.-P.); belia.garcia@bellvitgehospital.cat (B.G.-P.); 2 Neurology Service, Epilepsy Unit, Hospital Universitari de Bellvitge-IDIBELL, Universitat de Barcelona, 08908 L’Hospitalet de Llobregat, Barcelona, Spain; jsalap@bellvitgehospital.cat (J.S.-P.); ghernandezp@bellvitgehospital.cat (G.H.-P.); mfalip@bellvitgehospital.cat (M.F.)

**Keywords:** qEEG, spectral analysis, spectrogram, seizures, rhythmic patterns, cyclic patterns, time domain, frequency domain

## Abstract

The electroencephalogram (EEG) is a cornerstone tool for the diagnosis, management, and prognosis of selected patient populations. EEGs offer significant advantages such as high temporal resolution, real-time cortical function assessment, and bedside usability. The quantitative EEG (qEEG) added the possibility of long recordings being processed in a compressive manner, making EEG revision more efficient for experienced users, and more friendly for new ones. Recent advancements in commercially available software, such as Persyst, have significantly expanded and facilitated the use of qEEGs, marking the beginning of a new era in its application. As a result, there has been a notable increase in the practical, real-world utilization of qEEGs in recent years. This paper aims to provide an overview of the current applications of qEEGs in daily neurological emergencies and ICU practice, and some elementary principles of qEEGs using Persyst software in clinical settings. This article illustrates basic qEEG patterns encountered in critical care and adopts the new terminology proposed for spectrogram reporting.

## 1. Introduction

Hans Berger recorded the first electroencephalogram (EEG) in 1924, 100 years ago [1]. It remains essential for the diagnosis, management, and prognosis of selected patient populations [2,3,4,5]. An EEG is the recording of the spontaneous electrical activity of the brain over time. This activity is thought to be due to the summation of excitatory and inhibitory postsynaptic potentials in the neurons of the superficial layers of the cerebral cortex. This activity is influenced by subcortical structures [5,6]. The EEG’s strengths lie in its high temporal resolution, real-time cortical function assessment, and bedside applicability [6].

With the advent of new digital EEG machines, long-term EEG recordings have become much more available. The recording of EEGs over extended time periods (hours to days) is called a continuous EEG (cEEG). In recent years, these prolonged recordings, particularly in intensive care unit (ICU) settings, have become crucial for managing neurocritical patients. Over the past 20 years, substantial evidence has emerged highlighting the high incidence of undetected epileptic seizures in neurocritical patients, the correlation between EEG patterns and patient prognosis, and the potential for detecting brain ischemia. cEEG monitoring has greatly improved our ability to detect epileptic seizures and other potentially harmful EEG patterns, leading to the enhanced management of acute neurological conditions. However, the large volume of data generated and the complexity of EEG interpretation have posed significant challenges in terms of personnel and technical resources [5,7,8,9,10,11].

In this sense, the use of mathematical algorithms to quantify EEG signals has significantly aided in the interpretation and management of prolonged EEG data. Quantified EEG (qEEG) analysis, achieved through the mathematical processing of EEG signals, is increasingly utilized in clinical settings [5,7,8,9,10,11]. Numerous software toolboxes, such as FieldTrip, EEGLAB, and MNE-Python, are available to facilitate this process. Additionally, there are clinically validated and approved software tools, like Persyst^®^ (Persyst Development Corporation, Solana Beach, CA, USA), which have been widely used in recent years for EEG analysis.

Quantitative EEG refers to the application of any mathematical and analytical algorithms to characterize, transform, and compress raw EEG signals usually into a graphical representation [8,12,13,14], with many utilities in daily clinical practice and in research in a broad area of the neurosciences, including disorders of conscience of many etiologies, epilepsy, neurodegenerative diseases, or psychiatry, among others.

However, a challenge in the expansion of qEEGs lies in the diversity of analytical methods and software available. In this context, commercially available, clinically approved software has been crucial for advancing the use of qEEGs. Nowadays, the use of qEEGs in daily critical care practice is increasing [8,15,16,17,18]. For example, Persyst software provides qEEG panels designed for clinical settings that include visually simplified compressed displays summarizing various characteristics of the raw EEG. This allows clinicians to view many hours of compressed EEG data on a single screen, in contrast to the 10 or 20 s of raw EEG typically displayed per screen. Noteworthy, the use of qEEGs guides the review of many hours of raw EEG data highlighting epochs of special interest, making EEG revision more efficient and efficacious.

Furthermore, qEEGs aid in quantifying the burden of some events such as seizure, or ictal–interictal continuum patterns [19,20]. In addition, some nuances of background changes progressing over time, such as cyclic seizures or cyclic alternating pattern of encephalopathy (CAPE), can be easily recognized using qEEGs. However, it is important to keep in mind that due to this reduction in data, qEEGs in critical patients should be evaluated in conjunction with the raw EEG to avoid missing events, particularly those involving small-magnitude changes [14].

This paper provides an overview of the current applications of qEEGs in daily neurological emergencies and ICU practice, and some elementary principles of qEEGs using Persyst in clinical settings. The aim of this paper is to illustrate basic qEEG patterns encountered in critical care and adopt the new terminology proposed for spectrogram reporting using Persyst software [11,15,16]. qEEG techniques used in research or in other areas of neurosciences are out of the scope of this review.

## 2. Technical Background

Although highly automated software, such as Persyst, are available for quantification of EEG recordings, understanding the mathematical principles behind signal processing is essential. This knowledge helps one to grasp the utilities and limitations of qEEGs, enabling the customization of trends and panels to adapt to specific clinical situations encountered in daily practice.

Each graphic representation of an EEG characteristic over long time periods is referred as a ”trend” [5,13], and a setting of trends is called a qEEG panel. Typically, qEEG panels display time on the *x*-axis and other variables on *y*-axis, sometimes incorporating a third dimension in a color code, the *z*-axis.

### 2.1. Time Domain

The conventional EEG signal is an oscillatory wave displayed in the time domain on the *x*-axis (15 or 30 mm/s) and electrical activity in voltage units (μV) on the *y*-axis. Quantification in this time domain is related to the amplitude. Typically, a few seconds of EEG signal are analyzed, extracting data related to amplitude in this period of time (epoch), such as the maximum and minimum amplitude, the mean amplitude, the median amplitude, the peak amplitude, or the percentage of time that the EEG is suppressed [13,14].

These data are pixelated, with each pixel representing summarized amplitude information over a few seconds, allowing for a depiction in the timeframe of hours, so changes in amplitude over long periods of time are better recognized. In time domain trends, the *y*-axis remains related to amplitude.

The main time domain trends include the amplitude-integrated EEG (aEEG), envelope trend analysis, and suppression percentage.

**Figure 1:**
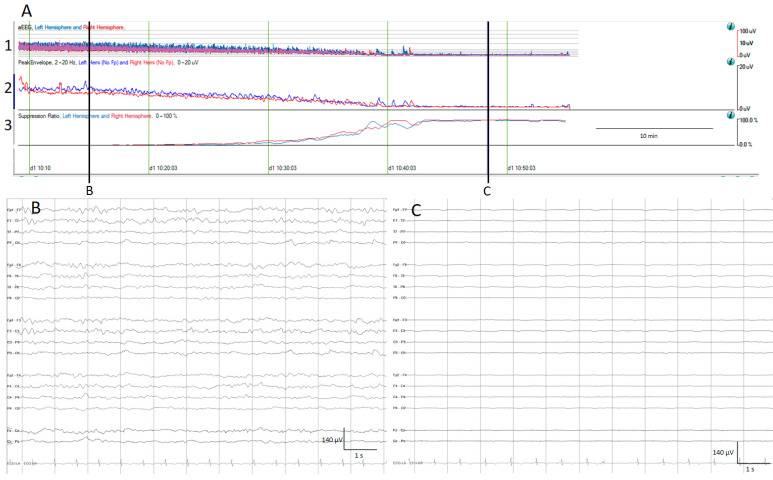
(**A**) qEEG amplitude measures. For all qEEG measures, the left hemisphere is depicted in blue and the right hemisphere in red; time is represented on the *x*-axis and amplitude in different scales on the *y*-axis. 1. Amplitude integrated EEG (aEEG): the maximum and minimum amplitude of each epoch connected with a vertical line is depicted using a semi-logarithmic scale (linear from 0 to 10 µV, and logarithmic from 10 to 100 μV). 2. Envelope trend: the peak amplitude from each epoch is plotted. 3. Suppression percentage: shows the percentage of suppression ranging from 0% (no suppression) to 100% (complete suppression). At the beginning, the raw EEG recording (**B**) and all amplitude EEG trends show normal amplitudes, and progressively the amplitude decreases as is depicted by the drop on the aEEG, peak envelope, and rise in suppression percentage. At the end of the recording, there is a complete suppression of the EEG (**C**).

The aEEG is a trend which has been used for a quite long time in neonatologist units [21]. After filtering, rectifying, and smoothing the raw EEG, the maximum and minimum amplitude for each epoch connected with a line is plotted on the *y*-axis, using a semi-logarithmic scale (linear from 0 to 10 μV, and logarithmic from 10 to 100 μV); practically, this means the low amplitudes are magnified [13,14] (Figure 1).

The envelope trend is another amplitude-related trend; unlike the aEEG, the envelope trend only plots one value for each epoch, either the median amplitude (median envelope trend) or the peak amplitude (peak envelope trend). While the median amplitude is less susceptible to artefacts, it may overlook small amplitude seizures, contrary to the peak envelope trend [13].

The suppression percentage indicates the percentage of the time EEG is suppressed within an EEG epoch; for this purpose, usually the detection of suppression is setup to an amplitude less than 5 μV for more than 0.5 s. In Persyst software (In this present work, versions 13 and 14 were used), this setup can be customized. Therefore, suppression percentages range from 0%, indicating continuous cerebral activity, to 100%, indicating complete suppression [13] (Figure 1).

### 2.2. Frequency Domain

**Figure 2:**
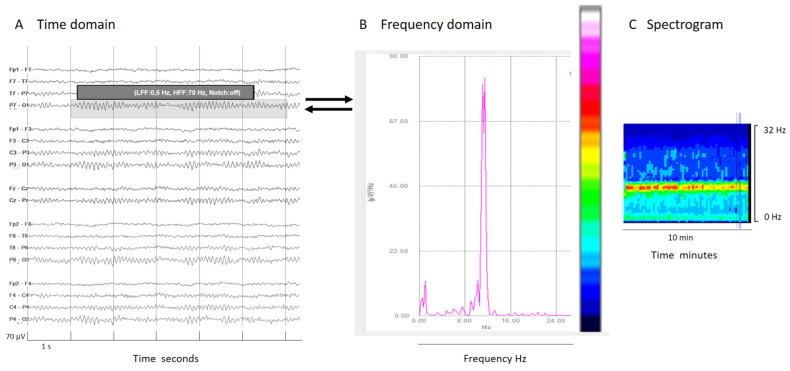
(**A**) An EEG shown in a classical EEG visualization; the time domain: *x*-axis 1s/division scale and *y*-axis 70 μV/division. (**B**) Fourier transform of a segment of an EEG (shadow rectangle on (**A**)). (**C**) Spectrogram: a color scale is applied to the Fourier transform (from white and warm colors for higher power to cooler colors (black) for lower power) and time is represented on the *x*-axis in minutes or even hours, with frequency on the *y*-axis and color on the *z*-axis. Each epoch of Fourier analysis becomes a column of pixels. In this normal EEG recording, the posterior dominant alpha rhythm produces a peak of power at 10 Hz in the Fourier transform; this is clearly visible on the color code as a red–yellow line at 10z in the spectrogram, indicating the maximal power at that frequency.

Frequency domain tools are based in the analysis of the contribution of each frequency to an EEG signal epoch. By applying Fourier transform, conventional EEG signals (time domain) are decomposed into frequency components (frequency domain, see Figure 2). The frequency (Hz) of the signal is plotted on the *x*-axis and power, defined as the area under the Fourier spectrum amplitude curve within a frequency value, measured in μV^2^ is plotted on the *y*-axis. Essentially, this represents the contribution of each frequency to the overall signal power. With some further mathematical and analytical techniques, many useful qEEG tools emerge, such as color density spectral array (CDSA, also known as spectrogram), absolute power, relative power, power ratio, spectral edge frequency, asymmetry spectrogram, and rhythmicity spectrogram [11,13,14,22].

Some frequency domain tools depict another frequency characteristic of the EEG signal using a color code (the *z*-axis).

The spectrogram, the most popular qEEG measure, plots time on the *x*-axis, frequency on the *y*-axis, and power within the frequency band on the *z*-axis using a color code. Usually, warm colors (white, red, yellow) represent high power of a frequency in their contribution to the whole epoch signal, while cool colors mean small or no contribution to the overall EEG signal (Figure 2).

**Figure 3:**
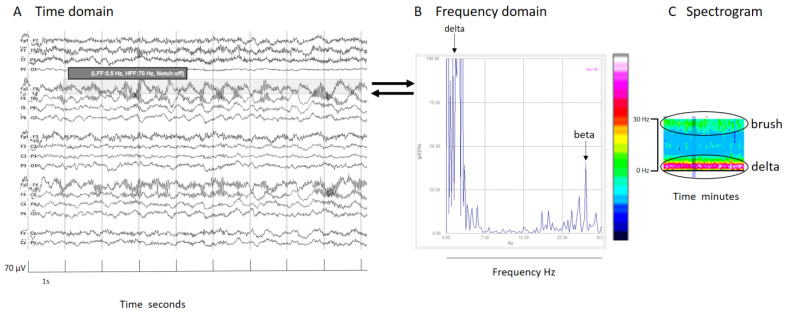
(**A**) An EEG shown in the time domain. (**B**) Fourier transform of a segment of an EEG (shadow rectangle on (**A**)) with a delta brush; clearly recognizable is a main peak of power at delta frequencies that arrives at red colors in the color scale, and other peak at beta frequencies that arrive at green colors in the color scale. (**C**) Spectrogram with a clear increase of power in the delta band (red band) and a less pronounced increase of power (green band) in beta frequencies that conform to the brush. It is interesting to note that if the frequency analysis had been cut at 20 Hz, the peak of the beta component (27 Hz, in this specific case) would have been lost. It is important to know at least some mathematical details of the analysis to apply it properly in clinical practice. For all details about this case, see Supplementary Figure S1.

In signals mainly composed by two prevalent frequencies, like extreme delta brush, the spectrogram illustrates each frequency’s contribution. Schmitt and colleagues described this pattern in 30% of NMDA encephalitis patients as a rhythmic delta activity at 1–3 Hz with superimposed bursts of rhythmic 20–30 Hz beta frequency activity “riding” on each delta wave [23]. It is crucial to know and specify the frequency range analyzed. In this specific case, because the superimposed beta frequency is above 20 Hz, restricting the analysis to frequencies up to 20 Hz would mean its presence would go unnoticed (Figure 3).

There are other trends arising from Fourier transformation. The absolute power is the area under the Fourier spectrum amplitude curve across all frequency ranges. Also, it is possible to calculate the power of certain frequencies of interest in a specific clinical situation. The usual measures are delta power, theta power, alpha power, or beta power [13]. Even though the frequency bands are well defined in the EEG glossary (delta (delta 0.1–< 4 Hz, theta4–< 8 Hz, alpha 8–13, and beta >13–30 Hz) [24], there are some variations in methodology and different authors use slightly different boundaries to calculate the power in these frequency bands [13,25].

**Figure 4:**
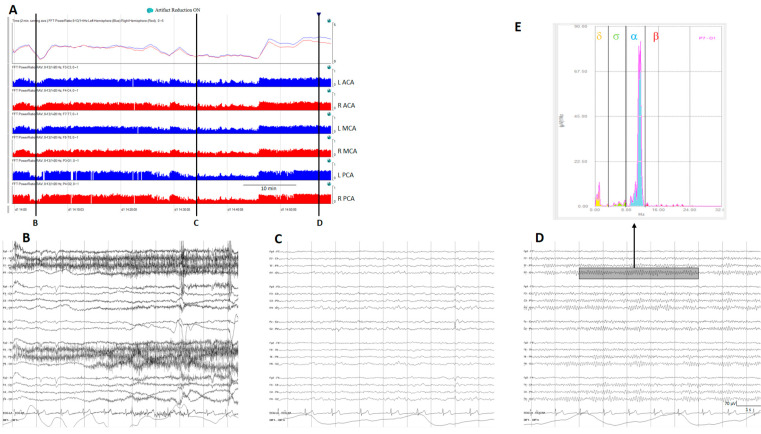
(**A**) A qEEG panel from a normal subject; from top to bottom: ADR (alpha-to-delta ratio) from the right and left hemispheres; RAV (relative alpha variability) from the major vascular territories: anterior cerebral artery (ACA), middle cerebral artery (MCA), posterior cerebral artery (PCA). (**B**) Awake with eyes open, (**C**) sleeping, and (**D**) awake with eyes closed. Note the variability on the ADR and on the RAV in a normal subject. (**E**) Fast Fourier transform from a period of EEG (rectangle on (**D**)); the power of each frequency band is shown in a different color. To calculate ADR, the power of alpha frequencies (8–13 Hz) is divided by the power of delta frequencies (1–4 Hz). To calculate RAV, the alpha power (8–13 Hz) is divided by the total power (1–20 Hz). ADR: alpha-to-delta ratio. RAVL relative alpha variability. L: left, R: right.

Relative power, another time domain measure, calculates the ratio of power for a specific frequency of interest to total power; the most used is relative alpha variability (RAV), assessing variability in alpha power relative to total power. In normal subjects, RAV exhibits significant variability (see Figure 4), which diminishes in patients at risk of delayed cerebral ischemia (DCI) after aneurysmal subarachnoid hemorrhage (aSAH). In this situation, RAV is visually graded from 4 to 1. Four means excellent variability with excursions (variability) from baseline occurring once per hour or of greater than 15% (visually has the appearance of skyscrapers), 3 is good (excursions of 10% at least every 4 h), 2 is fair (only small or infrequent excursions), and 1 is poor (no excursions greater than 2%) [26].

The power ratio is one of the trends that generates more interest in evaluating aSAH patients at risk of DCI. Power ratio refers to the ratio of power of two different frequency bands; for example, the alpha-to-delta ratio (ADR). Many other ratios between frequency bands are feasible and in some clinical situations useful [13,25,27].

The frequency below which a certain percentage of total power is located is called spectral edge frequency (SEF). On ICU monitors, SEF95 is commonly calculated, indicating the frequency below which 95% of the total power resides [13].

Another critical aspect emphasized with qEEGs is EEG symmetry. The asymmetry index is a two-dimension trend plotting time on the *x*-axis, and percentage of asymmetry on the *y*-axis [28]. Asymmetry spectrogram, a three-dimensional trend, displays time on the *x*-axis, frequency on the *y*-axis, and percent asymmetry on the *z*-axis (color code). Soft colors denote less asymmetry, while darker colors indicate greater asymmetry, with blue colors typically representing more power in the left hemisphere and red in the right hemisphere [28,29].

The rhythmicity spectrogram [13,29] is a proprietary trend developed by Persyst^®^ (Version 13 and 14), highlighting rhythmicity. Time is displayed on the *x*-axis, frequency on the *y*-axis, and power in frequency components with a high degree of rhythmicity in a color scale from low rhythmicity and low power (yellow) to high rhythmicity and high power (blue).

#### Nomenclature of Spectrograms

Some authors have proposed a nomenclature for spectrogram EEG patterns to facilitate communication, standardization, and training. They define the following categories: solid or regular flames, choppy or irregular flames, broadband monotonous, narrowband monotonous, suppression or low power, and stripes.

It is advisable not to rely solely on the spectrogram but to review all changes across the entire qEEG panel and correlate them with raw EEG features.

**Figure 5:**
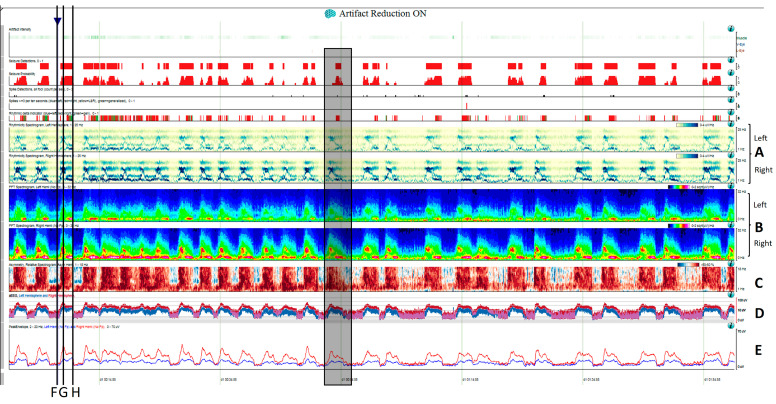

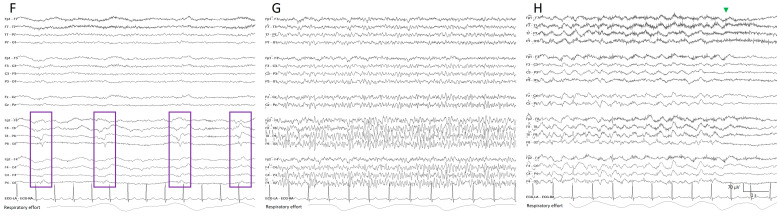
Two hours of a qEEG panel showing multiple focal seizures with the appearance of “solid flames”, one of which is highlighted with a shadow rectangle. (**A**) Rhythmicity spectrogram: *y*-axis frequencies from 1 to 25 Hz, showing an increase of rhythmicity (darker blue) in high frequencies at the beginning of the seizure that evolves to more rhythmicity in the lower frequencies at the end of the seizure, clearly more pronounced in the right hemisphere. (**B**) The spectrogram color scale represents power. Warmer colors (white–red) represent higher power and cooler colors (blue) represent lower power for each frequency band from 0 to 32 Hz (*y*-axis). Each seizure is a red–yellow flame more prominent in the right hemisphere. (**C**) Relative asymmetry spectrogram: a color scale represents power asymmetry in percentages between pair of homologous channels in both hemispheres at each frequency (*y*-axis from 1 to 18 Hz). White represents no asymmetry and the degree of darkness reflects more asymmetry until 50%; red marks more power in the right hemisphere and blue in the left hemisphere. In this case, there is a clear asymmetric increase in power in the right hemisphere during the seizures and because there is the presence of slow waves between seizures in the right hemisphere also, and asymmetry with an increase in power in the delta band is seen between seizures. Both amplitude trends, (**D**) amplitude integrated EEG (aEEG) and (**E**) peak envelop, show a consistent increase in amplitude during seizures, more accentuated in the right hemisphere. In this specific patient, the automated seizure detector recognizes the seizures; 10 s of raw EEG at (**F**) slowing in the background frequency, with the presence of lateralized periodic discharges (LPDs) over the right hemisphere at 0.4 Hz, with a plus modifier (superimposed fast activity). This pattern belongs to the ictal–interictal continuum. A total of 10 s of raw EEG at (**G**) continuous fast frequencies with spiky morphology with more amplitude in the right side, and diffusion to the left hemisphere. A total of 10 s of raw EEG at (**H**) the seizure has evolved, and rhythmic delta activity is present with superimposed spikes and sharp waves until the seizure abruptly finishes (green arrow). From Veciana and colleagues, 2024 [30] with permission.

**Figure 6:**
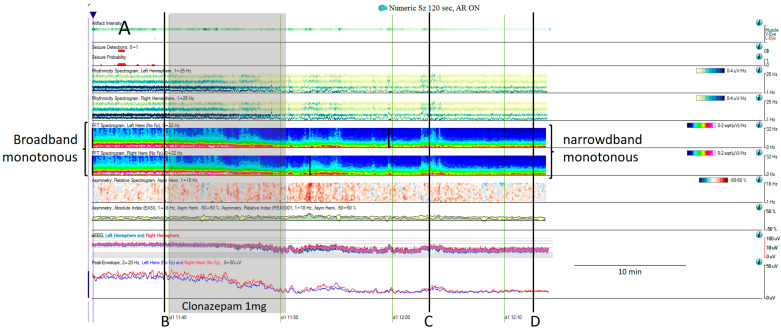

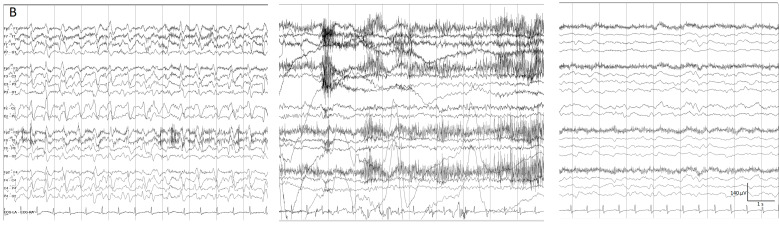
(**A**) One hour of a qEEG panel in a 67-year-old man with a past medical history of acute myeloid leukemia and renal failure. He was admitted with a fever (treated with cephepime) followed by confusion. The first part of the recording shows the presence of a broad band monotonous on the spectrogram and an increase in amplitude without asymmetry that matches with the raw EEG at (**B**) generalized periodic discharges at 1.7 Hz; this pattern belongs to the ictal–interictal continuum. The patient was confused and not following commands. Anti-seizure medication was administrated (intravenous clonazepam 1 mg, shadow rectangle). The raw EEG at (**C**) belongs to a period of patient clinical examination with muscle and movement artefacts on the EEG recording. Intermixed with the artefact, an improvement in the background EEG activity could be noticed. At that point, the patient followed commands. The raw EEG at (**D**) shows background slowing with no epileptiform discharges. The trend now shows a narrowband monotonous on the spectrogram, less amplitude on the aEEG, and peak envelope trends. The patient fulfils the criteria of nonconvulsive (electroclinical) status epilepticus with a clinical and EEG improvement of an ictal–interictal continuum pattern after anti-seizure medication. The rise in power and amplitude observed in the qEEG panel matches with artefacts as it is shown in (**C**).

**Figure 7:**
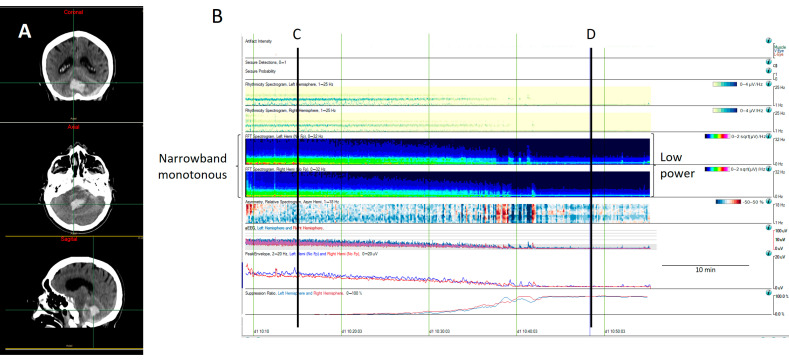

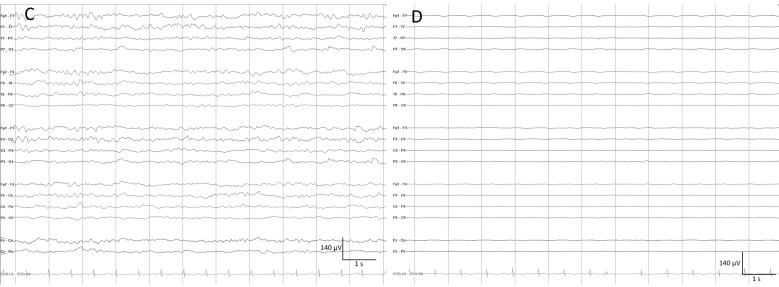
A computed tomography (CT) scan from an 81-year-old man with a cerebellar hematoma (**A**). (**B**) The quantitative EEG shows a progressive decrease in cerebral activity that it is revealed by a decrease in the power spectrum, changing from green (narrowband monotonous) to dark blue (lower power), as well as a decrease in the amplitude, as can be observed on the aEEG and envelope amplitude, along with an increase in the percentage of suppression. (**C**) The raw EEG at (**C**) shows a normal amplitude with the anterior–posterior gradient reversed. (**D**) The raw EEG at (**D**) shows a suppression of EEG activity corresponding to a low power spectrogram (dark blue), low amplitude on the aEEG and envelope train, and high suppression percentage. The patient was pronounced brain dead following confirmatory testing.

**Figure 8:**
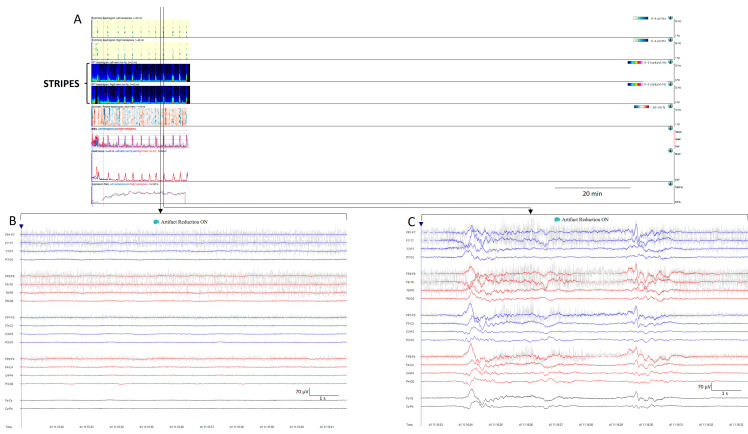
(**A**) Thirty minutes of a qEEG panel from a 71-year-old comatose man after resuscitation from a cardiac arrest. There are clear visible stripes on the qEEG trends, alternating between diffuse low power corresponding to suppression periods (raw EEG at (**B**)) and high power vertical stripes corresponding to bursts (raw EEG at (**C**)). On the raw EEG, the artefact reduction tool has been activated (ON) to remove the electromyography artefact (shadow grey in the raw EEG); then, EEG activity is clearly visible.

- Solid or regular flames is a spectrogram pattern characterized by an abrupt increase in power that stands out clearly from the background across a range of frequencies with the characteristic red and yellow colors indicating high power values. It resembles a candle flame with smooth edges, making it the most recognizable seizure pattern [11,15,16,28] (Figure 5).

- Choppy flames or irregular flames also show abrupt increases in power but have a more irregular appearance and tend to be less stereotyped than solid flames [15,16]. These patterns are more likely related to state changes or alternating patterns than to electrographic seizures, although seizures can occasionally manifest in this manner as well [11].
- Broadband monotonous represents a sustained high power with characteristic white, red, and yellow colors across a broad range of frequencies, exciding 5 Hz of bandwidth. In a raw EEG, it correlates with long periods of unchanging status epilepticus or periodic discharges, characterized by prolonged, high-amplitude activity [11,15,16] (Figure 6).

- Narrowband monotonous refers to a spectrogram with power spectrum relatively restricted to low frequencies (less than 5 Hz) with minimal variation in power; it is typically encountered in patients with encephalopathy [11,16]. Both broad and narrowband monotonous can persist unchanged over long periods or exhibit gradual changes that may or may not have a clear onset or resolution, sometimes reflecting spontaneous changes and at other times response to treatment (Figure 6).
- Suppressed or low power EEGs appear in spectrograms as a continuous low power represented typically by dark blue colors [11,15,16] (Figure 7).

- Stripes refers to a pattern characterized by the appearance of vertical stripes, where low power (background suppression) alternates with high power (bursts of activity), indicating intermittent periods of activity [11,16] (Figure 8).

## 3. Main Clinical Applications

This paper focuses on emergency and ICU patients. In these settings, qEEGs facilitate the reading and review of prolonged EEGs, also showing the possibility of the rapid detection of seizures and state changes. Using the capabilities of the most widely available software, the main clinical applications of the qEEG include the detection of seizures and status epilepticus, guiding the intensity of their treatment; the characterization and evolution of the ictal–interictal continuum (IIC) patterns and cyclic patterns; and the identification of patients with aSAH who are at high risk of DCI [5,8].

It is both possible and beneficial to apply qEEGs to EEG recordings of any duration. It aids in emphasizing aspects of EEG activity such as changes in background activity over long periods of time, the presence of cyclic or alternating patterns, and the detection of seizures, as well as the calculation of pattern burden. In addition, it has been identified that the use of certain qEEG trends reduces review time by 78% with minimal loss of sensitivity compared to conventional raw EEG review [31].

### 3.1. Seizure and Status Epilepticus Detection and Their Response to Treatment

The most common use of the qEEG is the detection of seizures and status epilepticus, calculating seizure burden, and identifying changes related to treatment response [8].

According to critical care EEG terminology, seizures are divided into two categories: electrical seizures and electroclinical seizures. Electrical seizures can only be reliably recognized with an EEG. Electroclinical seizures encompass clinical and electrical changes. Electrical seizures are characterized by epileptiform discharges averaging more than 2.5 Hz for 10 s or longer, or any pattern of the same duration with a definite evolution. Electroclinical seizures are time-locked EEG patterns of any duration with a definite clinical correlate or an EEG and clinical improvement with a parenteral antiseizure medication [4,20,32,33]. A seizure lasting longer than 10 min qualifies as status epilepticus, while in patients with numerous seizures lasting less than 10 min, a seizure burden exceeding 20% of any 60 min period (i.e., 12 min per hour) also qualifies as status epilepticus. An exception is an ongoing seizure with tonic–clonic activity, which only needs to last more than 5 min to qualify as status epilepticus [2,20,33,34].

**Figure 9:**
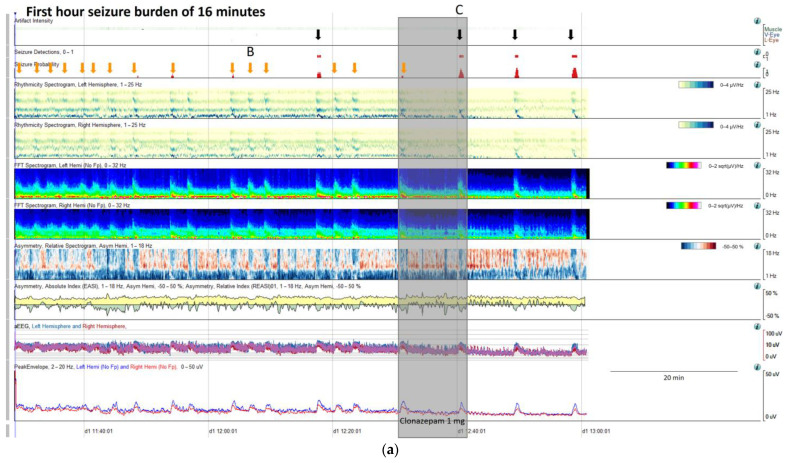

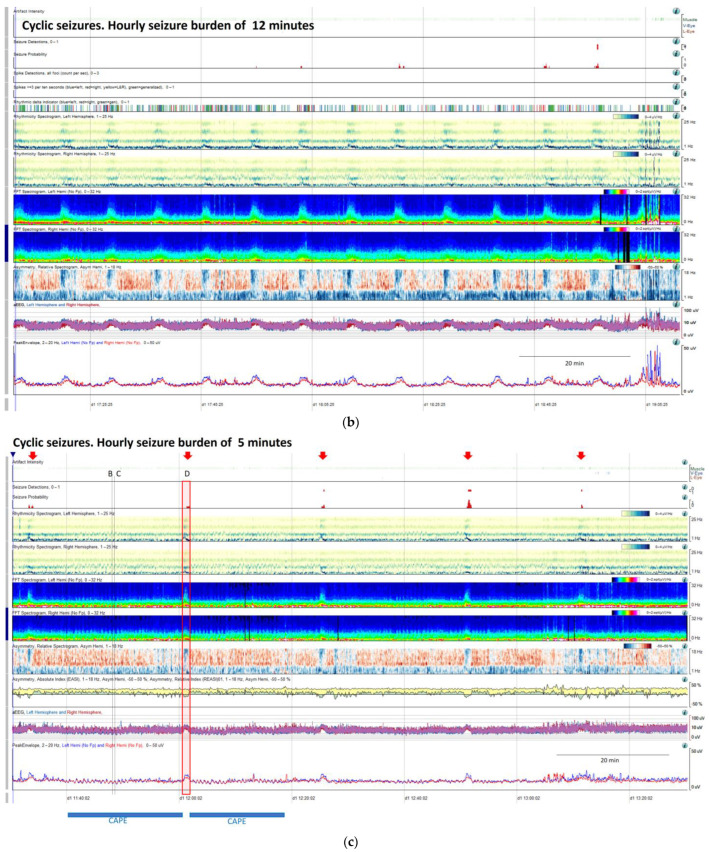
(**a**) qEEG in 2 h display from a 73-year-old man with past medial history of left ischemic stroke. In this panel is clearly visible, that seizure detector pointed 4 seizures (black arrows), however the seizure signature is clearly recognizable in many more occasions (orange arrows) that are not detected by the algorithm, but clearly recognizable using all the qEEG trends, cheeking the raw EEG all the arrows turn out to be seizures (raw EEG at (B) on Supplementary Figure S2 and matching clinically with eye version to the right and nystagmus. Rhythmicity spectrogram show a clear increase in high frequency bands at the beginning of the seizure that move to slow frequency bands, always more pronounced in left hemisphere. Spectrogram depicted a solid flame in the left hemisphere a little bit recognizable also in the right hemisphere. Asymmetry spectrogram is quite interesting; all the time shows a great power in low frequencies in the left hemisphere, on the raw EEG correlates with delta waves in that hemisphere, and during the seizures also an increase of left power in high frequency bands. aEEG and peak envelope depicted the typical arch shape in each seizure that tell us about and increase in amplitude during the seizures. In Supplementary Figure S2 you can find the raw EEG for the seizure pointed at (C). Note that after the intravenous antiseizure medication (ASM) administration the seizures separate and became better defined in evolution (a quite common situation in critical patients) therefore, easily recognizable by the automated seizure detector. First hour seizure burden was 16 min fulfilling the criteria of status epilepticus. (**b**) After the administration of lacosamide seizure burden decreases to 12 min per hour and seizures become cyclic. (**c**) Valproate was added and seizure burden further decrease to 5 min per hour, however a cyclic alternating pattern of encephalopathy also appear (blue line, raw EEG is shown in Supplementary Figure S2. Note the difference between cyclic alternating pattern more arch shape on the spectrogram (blue line) and seizures (red arrows) more triangular shape and smooth edges. Correlation with the raw EEG at (B) and (C) (CAPE), and at (D) (seizure) are shown in the Supplementary Figure S2.

Evolution is a cornerstone in seizures [2,20,32,33,34]. It is defined as to two unequivocal changes in frequency morphology or location. Evolution in frequency is highlighted by qEEGs, especially spectrograms or rhythmicity spectrograms. The typical spectrogram feature in seizures is the so-called “solid flame”, characterized by a triangular shape with smooth edges, reflecting a sudden increase of power (Figure 5) in the frequencies involved in the seizure [11,15,16,28,35]; in status epilepticus a broad band spectrogram can sometimes be observed, reflecting a high power across multiple frequencies (Figure 5 and Figure 9) [11,15,16]. Rhythmicity spectrograms emphasize power in frequency components with a high degree of rhythmicity, which has been proven useful by some authors for detecting subtle seizures and differentiating them from artifacts [29].

Changes in morphology usually entail changes in amplitude; because of that, amplitude trends such as aEEG or envelope amplitude trends are sensitive indicators of seizures and have been used extensively in pediatric ICUs. Increases in amplitude are depicted as upward deflections in the amplitude trends [28,35,36].

Asymmetry spectrograms are sensitive to the detection of focal seizures showing an increase in power in the hemisphere involved in the seizure, and they also can show post-ictal suppression [35].

The accuracy of qEEGs in diagnosing seizures has been addressed in several studies, using different trends and panels, different patient populations (adults, pediatrics, EMU, ICU), different strategies to interpret qEEG results (automated seizure detection algorithms, review by non-experts, or by experts), and different gold standards, yielding quite variable results (sensibility ranging from 26% to 100%, specificity from 38% to 91%, and false alarm ratio from 0.04 to 6.4 per hour) [10,15,29,36,37,38,39,40,41,42,43,44,45].

Accordingly, as mentioned before, a qEEG panel addressed to seizure and status epilepticus detection could benefit from displaying the following trends: spectrograms, rhythmicity spectrograms, amplitude-related trends like aEEG or envelop trends, asymmetry spectrograms (that can be complemented with an absolute or relative asymmetry index), and automated seizure detection algorithms [28,29,35,45].

High-amplitude seizures and focal seizures that evolve bilaterally are easily identified with qEEGs. The false positive identification of seizures occurred more frequently in the generalized seizure group. In contrast, seizures that are more challenging to identify with qEEG approaches include low-amplitude seizures, which exhibit small changes compared to background amplitude, or seizures that occurs in recordings with periodic discharges. Additionally, low-frequency, short-duration and smaller spatial extent seizures are also easily missed on qEEG evaluation [35,44,45]. To address this problem, it is essential to evaluate qEEGs in conjunction with raw EEGs. Once the focal seizure has been identified on the raw EEG, trends may be customized to focus on displaying only this region rather than the whole hemisphere in order to detect changes related to this focal seizure [5].

Another confounding situation is the presence of blink or muscle artifacts, which may be mistaken for seizures. In order to address this problem, as already mentioned, the evaluation of qEEGs simultaneously with raw EEGs is decisive in avoiding these pitfalls. Additionally, these artefacts in qEEG panels are slightly different from seizures. These artifacts usually have a sudden appearance, lacking the typical evolution with diagonal edges that is characteristic of seizures, on all trends [5]. This evolution is especially recognizable on rhythmicity spectrograms, and on spectrograms where this appearance has been termed “solid flames” [15,16]. Also, artifact reduction algorithms assist in diminishing artifacts.

Hence, the use of qEEGs is recommended to guide directed raw EEG review [28,29,31,36,45] with the goal of finding a good balance between seizure detection, avoiding false alarms, and saving time, leading to quicker treatment adjustments.

Once a seizure is identified on an EEG recording, their characteristic signature in the specific patient needs to be characterized, and, if necessary, the trends can be customized in order to guide further cEEG revision, and then seizure burden, cyclic seizures, and response to treatment will be easily identified (Figure 9).

Seizure burden is defined as the total amount of recorded time spent seizing on a cEEG per hour [20]. An increase in seizure burden is related to worse outcomes in critically ill children [19,46] and in adults [47]. Quantitative EEGs facilitate the calculation of seizure burden and their changes over time, which is especially interesting in relation to their response to treatment.

Cyclic seizures are common in critically ill patients [48,49] and clearly recognizable with qEEGs. Nevertheless, this term is not included in the standardized critical care EEG terminology 2021. Cyclic seizures are defined as recurrent seizures occurring at nearly regular intervals with a frequency greater than three per hour for at least 1 h [48,49] (Figure 9b). Their prognostic significance is controversial [48,49], and their pathophysiology is not fully understood; cortical spreading depolarizations have been suggested to be implicated [50,51,52].

IIC patterns have a typical qEEG signature that is very useful for gauging their prevalence and their spontaneous interruptions. In a patient with clinical suspicion of non-convulsive status epilepticus (NCSE), if an IIC pattern lasts at least 10 min or occupies at least 20% of any hour of recording, it is considered synonymous of possible electrographic status epilepticus. To establish a diagnosis of definite NCSE, an EEG and clinical improvement with parenteral antiseizure medication administration is needed [2,20,33,53]. However, there is a lack of standardization to evaluate this response; to fill this gap, a group of experts put forward consensus-based recommendations to avoid the misinterpretation of a spontaneous interruption of an IIC pattern as being induced by medication [4]. Response to intravenous ASM is considered present when there is an EEG interval without IIC lasting three times the longest prior spontaneous IIC-free interval, if any, but lasting a minimum of one continuous minute. In non-comatose patients, the return to baseline background frequency or the appearance of previously absent normal features as posterior dominant rhythm or sleep spindles should also be taken into consideration (Figure 6). In comatose patients, those with unknown background frequency, or those with a systemic condition, this return, or improvement of the background, cannot be applied [4]. Finally, in these situations, functional neuroimaging may also be useful [54,55].

New quantitative approaches are in development. Increases in alpha and beta bands have been found to be increased in seizures and can help to differentiate between periodic patterns and seizures [56]. Based on the current hypothesis that even focal seizures are a network phenomenon that involve widespread neuronal connectivity, scalp coherence measures have detected seizures in EMU patients, where scalp EEG has been negative [57]. Additionally, qEEG analysis of the background activity in temporal lobe epilepsy patients have found lower alpha–delta and alpha–theta ratios in the affected temporal lobe areas [58].

### 3.2. Changes in the Background and Cyclic Patterns

In emergency and ICU patients, qEEGs are especially useful for detecting changes in the background EEG activity. Sometimes these changes are gradual, take minutes or even hours, and are better appreciated with qEEG, as is the case during rising intracranial pressure, changes related to the depth of sedation, changes in response to antiseizure medication, state changes, or cyclic patterns that are sometimes associated with encephalopathies.

qEEGs can be useful in detecting changes related to elevated intracranial pressure (ICP) [59]. Increases in ICP lead to decreases in cerebral perfusion, which subsequently leads to EEG changes. The EEG alterations observed in this situation are progressive slowing, loss of fast frequencies, and, as a consequence, a decrease in the alpha–delta ratio. If the situation gets worse, an increase in suppression percentage may appear.

Ventriculoperitoneal shunt malfunction, in patients with hydrocephalus and intracranial pressure fluctuation, can manifest as episodes of transient unresponsiveness that correlate with diffuse delta slowing on an EEG [60,61]. In acute patients with presumed ICP fluctuations, Cheyne–Stoke breathing and cyclic patterns on EEG have been recorded [60,62]. In patients with a progressive increase in ICP and herniation and a decrease in amplitude and power on an EEG leading to complete suppression have been observed [62] (Figure 7). Some EEG alterations can be detected even prior to clinical deterioration or neuroimaging changes [62,63], giving a window of opportunity to treat and reverse this situation.

**Figure 10:**
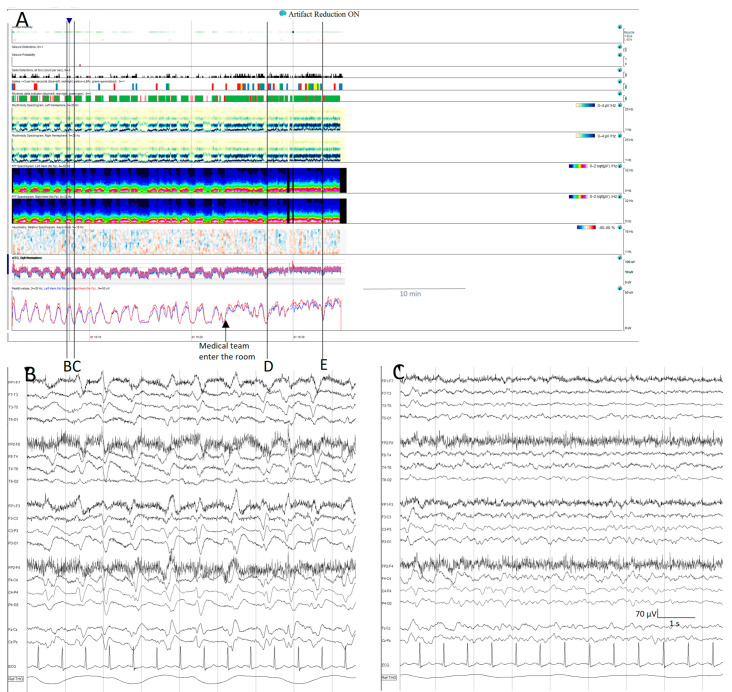
Cyclic alternating pattern of encephalopathy (CAPE): (**A**) cyclic alternating pattern is clearly visible on this EEG from an 82-year-old woman. There are like a series of arches on the qEEG trends, the top corresponding to a raw EEG at (**B**) showing generalized periodic discharges (GPDs) with triphasic morphology with amplitude around 100 µV, alternating with periods of theta background with amplitude around 50 µV (**C**). Cheyne-stokes respiratory pattern is correlated with the EEG changes, hyperpnea is present during high amplitude GPD and apnoea/hypopnea during low amplitude theta periods. When the medical team enter the room, the spontaneous alternating pattern shifts to a more amplitude and GPDs pattern, and the stimulation ((**D**,**E**) see Supplementary Figure S3D,E) induced the GPD pattern. GPDs pattern fulfils the criteria of IIC and in this specific patient, as it happens in many encephalopathic patients, correspond to the most stimulated state, despite the theta pattern looks like, apparently, more normal.

On some occasions, background patterns alternate periodically. In the latest guidelines from the American Society of Neurophysiology, this presence of alternating patterns, typical of encephalopathy, has been defined as an entity under the term CAPE. It is defined as a spontaneous alternation of two background patterns, each of them lasting at least 10 s, in a regular manner for at least six cycles [20]. CAPEs have a characteristic signature on qEEGs; in the spectrograms of some patients, this can be seen as a series of arches (Figure 10).

In some patients, their pathological backgrounds are intermingled with seizures. Using qEEGs makes it easier to recognize changes in the background, and simultaneously identify prominent events, such as stimulus-induced changes, including reactivity, SIRPIDs (stimulus-induced rhythmic, periodic, or ictal-appearing discharges), or stimulus-terminated patterns [20,64,65], as well as seizures (Figure S3 Supplementary Materials, Figure 9).

### 3.3. Aneurysmal Subarachnoid Hemorrhage (aSAH)/Delayed Cerebral Ischemia (DCI)

In the setting of a patient suffering from aSAH, qEEGs have three main purposes. First, to allow the detection of seizures and status epilepticus, especially the non-motor forms, and to manage them according to the clinical and EEG response. Second, they can provide insights into the clinical prognosis of these patients. Lastly, they may assist in assessing the risk of impending DCI.

Clinical seizures may occur at any time in SAH patients: onset seizures occur at the time of hemorrhage, early seizures occur during the first week, late seizures occur after the first week during the hospital stay, and delayed seizures occur after hospital discharge [66,67,68]. Noteworthy, not all spells of abnormal movements in aSAH patients are seizures; for example, some tonic movements are related to increased ICP [59]. However, nonconvulsive seizures and NCSE have high incidences in these patients. Up to 7–18% of aSAH patients were diagnosed with electrical seizures during their hospital stay and 3–13% with NCSE [47,69,70,71,72]. A more suitable qEEG panel for seizure detection and management is described in Section 3.1 (seizure and status epilepticus detection and their response to treatment).

As mentioned, some EEG features found in cEEG monitoring have been related to prognosis. Periodic epileptiform discharges, electrographic status epilepticus, the absence of sleep architecture, and increased seizure burden are associated with unfavorable outcome [47,73]. NCSE has been identified as an independent predictor of unfavorable outcome in patients suffering aSAH, along with age, poor-grade SAH, and the presence of delayed ischemic neurological deficit [72]. In addition, the risk factors identified for developing NCSE are the presence of poor-grade SAH, older age, ventricular drainage, and cerebral edema on CT (computed tomography) [69]. Even when medical treatment successfully terminates SE, the long-term outcome remains poor [69,72]. However, a favorable outcome is possible in some patients. In the Vychopen cohort, NCSE was diagnosed in 19 patients (3.7%), with one achieving good prognosis [72].

Finally, one of the more emerging capabilities of qEEGs in daily clinical practice is assessing the risk of impending DCI.

DCI occurs in around 30% of aSAH patients, mostly between days 4 and 14 after aSAH [68].

DCI has been defined as the new occurrence of focal neurologic impairment or a decrease in the Glasgow Coma Scale of at least 2 points, persisting for a minimum of 1 h, not explained by other causes or the presence of cerebral infarction on structural brain imaging and not present within the first 48 h after aneurysm occlusion [74].

DCI is much more than vasospasm: not all patients with vasospasm develop DCI, and DCI can occur without vasospasm [75,76]. The physiopathology of DCI is complex and not fully understood, comprising impaired cerebral autoregulation, microcirculatory dysfunction, microthrombosis, cortical spreading depolarization [77,78,79,80], and neuroinflammation. All these factors contribute to the mismatch between metabolic supply and metabolic demand that generates DCI [68].

Changes in EEG activity related to cerebral blood flow reduction are decrease in rapid EEG activity, increase in slow frequencies, and finally, suppression [81,82,83]. The qEEG trends that pick up these alterations are a decrease of power in the alpha or beta bands and increase in power in the delta band. The way to magnify these changes are the ratios between the power of fast frequencies in the numerator and total power or slow frequency power in the denominator [26,27,73,84,85,86,87,88].

Although there is some heterogenicity between authors, the general idea is to monitor the major cerebral vascular territories. Different settings of electrodes have been used to cover the anterior cerebral artery (ACA), middle cerebral artery (MCA,) and posterior cerebral artery (PCA) of each hemisphere [26,85,87,88,89] (Table 1).

Many authors use the RAV visual score introduced by Vespa and colleagues, 1997 [26]. RAV was graded as excellent (4) good (3), fair (2), or poor (1) based on the differences between peak and trough. A decrease of one grade was considered significant. It is also possible to quantify RAV using the formula (Peak value − Trough value)/(Peak value + Trough value) [26].

**Figure 11:**
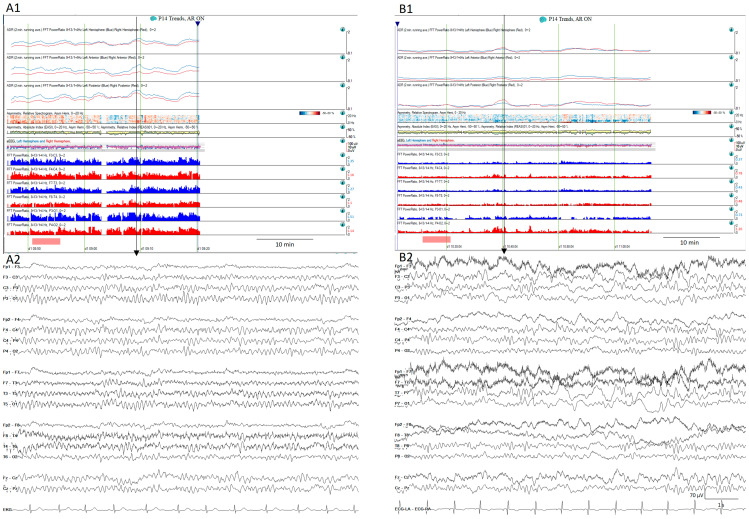
Normal (**A1**) quantitative trends and the (**A2**) raw EEG corresponding to the vertical arrow from a patient on day 2 after SAH. The same patient on day 6 after aSAH (**B1**) shows a decrease in the alpha-to-delta ratio in all territories, more pronounced in the left temporal regions. (**B2**) shows the raw EEG matching with the vertical arrow that corresponds to the moment after the stimulation showing slow waves more pronounced over the left hemisphere.

ADR is one of the qEEG trends that perform best for the detection of impending DCI [27,90]. ADRs have some variability in normal subjects at different levels of arousal, so the best way to compare ADRs in the same patient is to use the ADR after stimulating the patient, the so-called post-stimulation ADR [27]. This is considered a significant decrease > 10% from the baseline persisting for at least 6 consecutive hours, or at least 50% of decrease from the baseline in the post-stimulus state [27,85] (Figure 11).

In addition to the decrease in fast activity and increase in slow activity highlighted by some qEEG trends already mentioned, some studies identified the presence of seizures and epileptiform abnormalities, defined as sporadic epileptiform discharges and ictal–interictal continuum abnormalities (lateralized or generalized periodic discharges or lateralized delta activity), as a predictor of DCI [85,91,92,93]. Additionally, Kim and colleagues 2022 [92] found a higher burden of epileptiform discharges in a patient who developed DCI after aSAH, suggesting that the quantification of epileptiform discharges may be used as a biomarker to predict those at higher risk of developing DCI [92].

More interestingly, changes in EEG parameters usually precede clinical and neuroimaging changes by hours or even days [85,90,91,94,95], providing a window of opportunity for therapeutic interventions. Another clear advantage of EEGs is that they provide real-time continuous information about cortical activity in contrast to other techniques that evaluate blood vessel caliber or blood flow, which can at best be performed only once or twice a day.

For all these reasons, the 2023 guidelines from the American Heart Association/American Stroke Association for the management of aSAH patients recommended (class of recommendation: 2a, level of evidence: B) a continuous EEG to detect seizures and predict DCI in high-grade SAH [68].

As in many other areas of medicine, the prediction of the impending risk of DCI requires a multimodal approach [68,93]. The best option includes the use of EEGs, transcranial doppler ultrasounds (TCDs), clinical variables, neuroimaging techniques, and in high-grade aSAH even invasive monitoring of brain tissue may be considered [68,96].

## 4. Conclusions

The EEG is a non-invasive bedside technique that provides real-time continuous information about cortical activity. Nowadays, qEEG techniques add the advantage of displaying hours and even days of an EEG on a single screen in contrast to the only 10 or 20 s displayed by raw EEGs; more interestingly, a qEEG synchronized with video and a raw EEG can be displayed at the same time at the bedside in the ICU and allow for remote access, helping real-time evaluation by the neurophysiologist on call.

It is feasible to implement qEEGs in daily practice. Knowing some technical details and using clinically validated software helps to customize the qEEG trends to each clinical situation. The recognition of the fingerprints observed on qEEG trends of seizures, periodic and cyclic patterns helps to save time in the evaluation of long EEG recordings and increases diagnostic accuracy.

## 5. Future Directions

There is a need for technical guidelines, including regarding the setting of electrodes and all mathematical details of the processing of the EEG signal, to make results across different laboratories comparable and reproducible.

An initial qEEG terminology is being proposed in some studies; going further in standardizing qEEG terminology is essential for educational purposes, research, and clinical applications.

Going deeper into research on the utility of new and less broadly used mathematical treatment of the EEG signal, such as entropy, coherence, or synchronization, will bring some insight into the pathophysiology of some diseases and could help in the management of some patients.

## Figures and Tables

**Table 1:** Some settings of electrodes used to roughly cover major vascular territories.

		ACA	MCA	PCA
Vespa et al., 1997 [26]	L	F3-T3	T3-P3	P3-O1
	R	F4-T4	T4-P4	P4-O2
Muniz et al., 2018 [89]; Rosenthal et al., 2018 [85]; Balança et al., 2018 [88]	L	F3-C3	C3-T3	P3-O1
	R	F4-C4	C4-T4	P4-O2
Zheng et al., 2022 [87]	L	Fp1-F7, Fp1-F3	F7-T3, T3-T5, F3-C3, C3-P3	T5-O1, P3-O1
	R	Fp2-F8, Fp2-F4	F8-T4, T4-T6, F4-C4, C4-P4	T6-O2, P4-O2

L—left, R—right; ACA—anterior cerebral artery; MCA—middle cerebral artery; PCA—posterior cerebral artery.

## Supplementary Materials

The following supporting information can be downloaded at: https://www.mdpi.com/article/10.3390/brainsci14090939/s1.

## Abbreviations

ADR alpha-to-delta ratio; aEEG amplitude-integrated EEG; aSAH aneurysmal subarachnoid hemorrhage; ACA anterior cerebral artery; ASM antiseizure medication; CAPE cyclic alternating pattern of encephalopathy; CDSA color density spectral array; cEEG continuous EEG; CT computed tomography; DCI delayed cerebral ischemia; EEG electroencephalogram; EMU epilepsy monitoring unit; GPDs generalized periodic discharges; IIC ictal–interictal continuum; ICU intensive care unit; ICP intracranial pressure; LPDs lateralized periodic discharges; MCA middle cerebral artery; NCSE non-convulsive status epilepticus; PCA posterior cerebral artery; qEEG quantitative EEG; RAV relative alpha variability; SIRPIDs stimulus-induced rhythmic, periodic, or ictal-appearing discharges; SEF spectral edge frequency; TCDs transcranial doppler ultrasounds.
