# Supplementary material for: Utility of Quantitative EEG in Neurological Emergencies and ICU Clinical Practice
Source: Brain Sci. 2024 Sep 20;14(9):939. doi: 10.3390/brainsci14090939 (PMC11430096; doi:10.3390/brainsci14090939)
Supplement: Supplementary file 1 [file brainsci-14-00939-s001.zip › brainsci-3104843-supplementary.pdf]

Supplementary Figures

Supplementary Figure S1

Supplementary Figure S1A

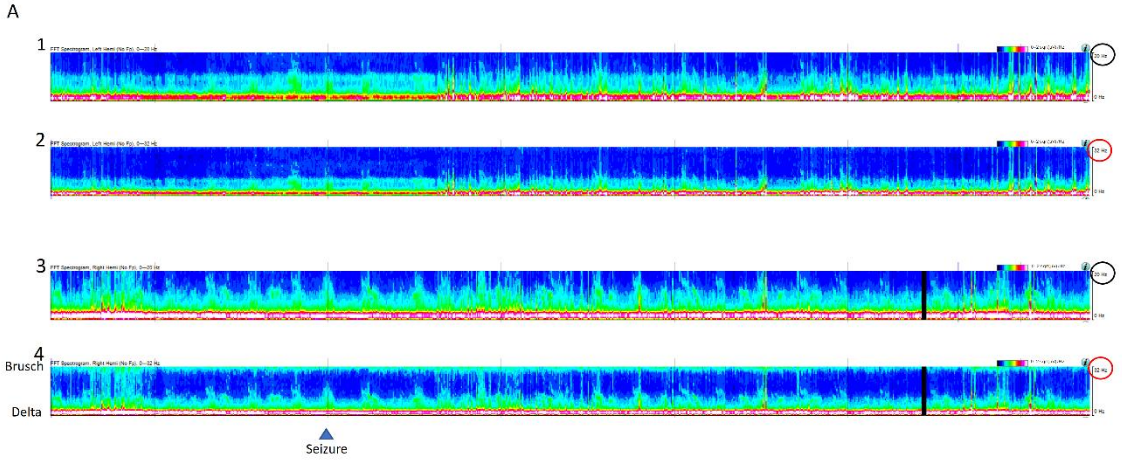

Supplementary Figure S1A. Two-hour spectrogram from an 18-years-old-man with a NMDA encephalitis with asymmetrical extreme delta brush. 1 spectrogram from the left hemisphere with the analysis range frequencies until 20 Hz by comparison 2 with the analysis range frequencies until 32 Hz. 3 spectrogram from the right hemisphere with the analysis of range frequencies until 20 Hz by comparison 4 with the analysis range frequencies until 32 Hz. The right delta brush is only visible with the analysis range until 32Hz as an increase of delta power (white and red band at the delta frequencies) and a green band at the beta frequencies (brush).

Supplementary Figure S1B

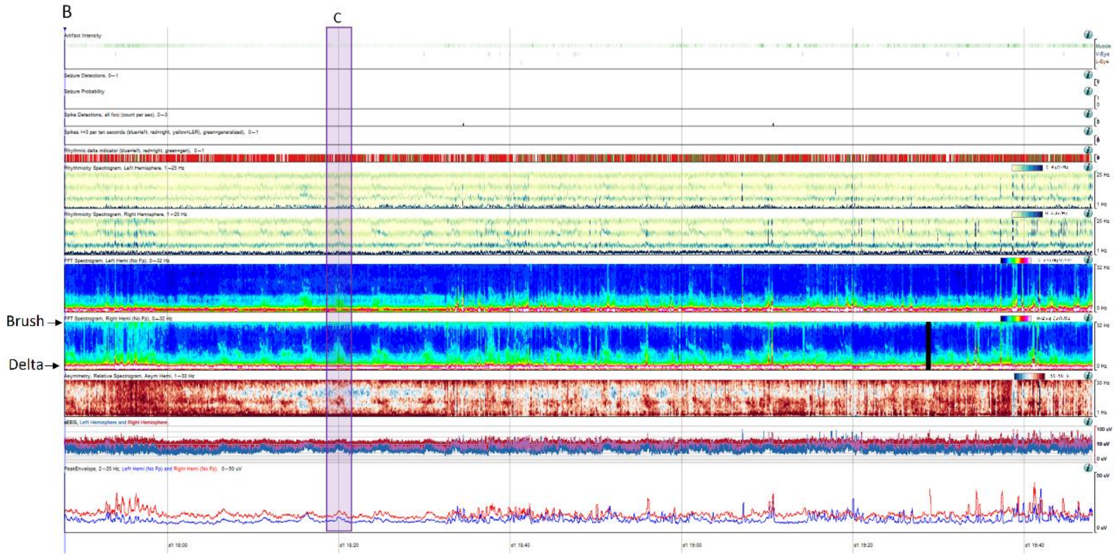

Supplementary Figure S1B. Two-hours qEEG panel with the right delta brush, and some right upward deflections, one highlighted with a purple box (C) matching with a right temporal seizure at the raw EEG shown in the next figures and highlighted with a black box.

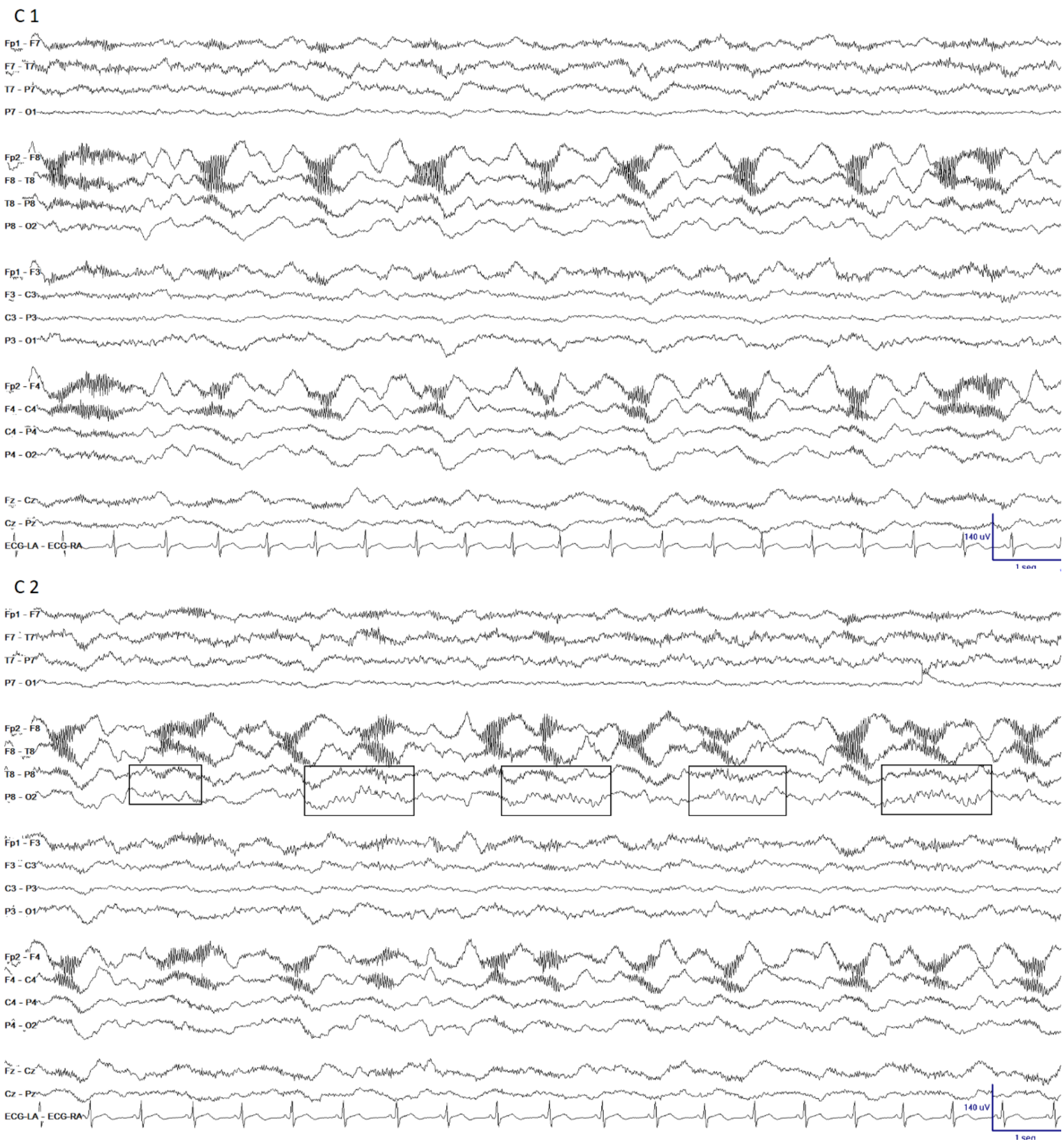

C 3

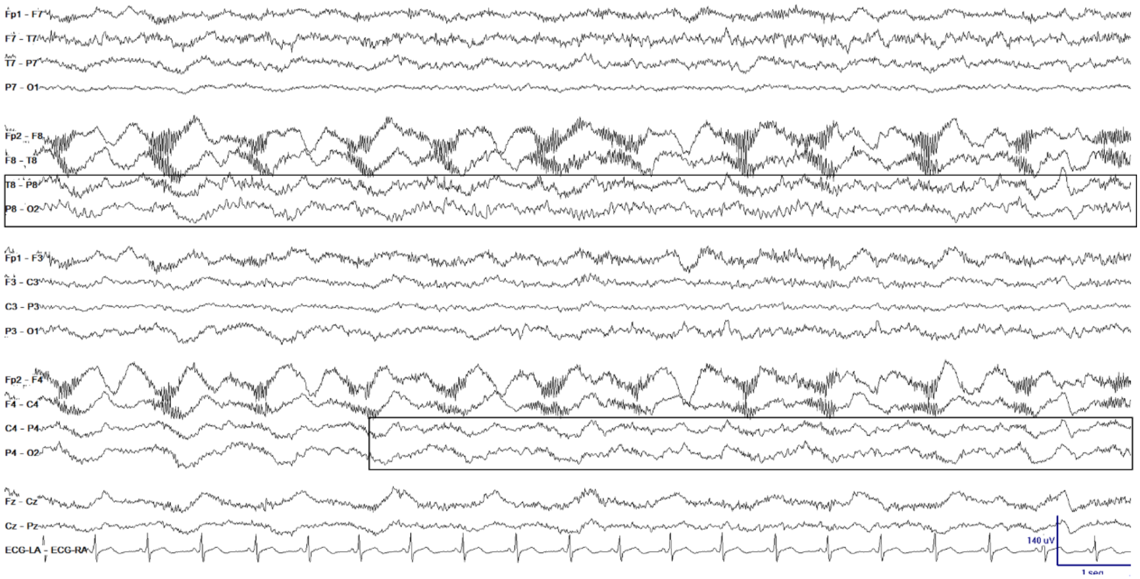

Fp1 - F7
F7 - T7
T7 - P7
P7 - O1
Fp2 - F8
F8 - T8
T8 - P8
P8 - O2
Fp1 - F3
F3 - C3
C3 - P3
P3 - O1
Fp2 - F4
F4 - C4
C4 - P4
P4 - O2
Fz - Cz
Cz - Pz
ECG-LA - ECG-RA
140 uV
1 sec

C 4

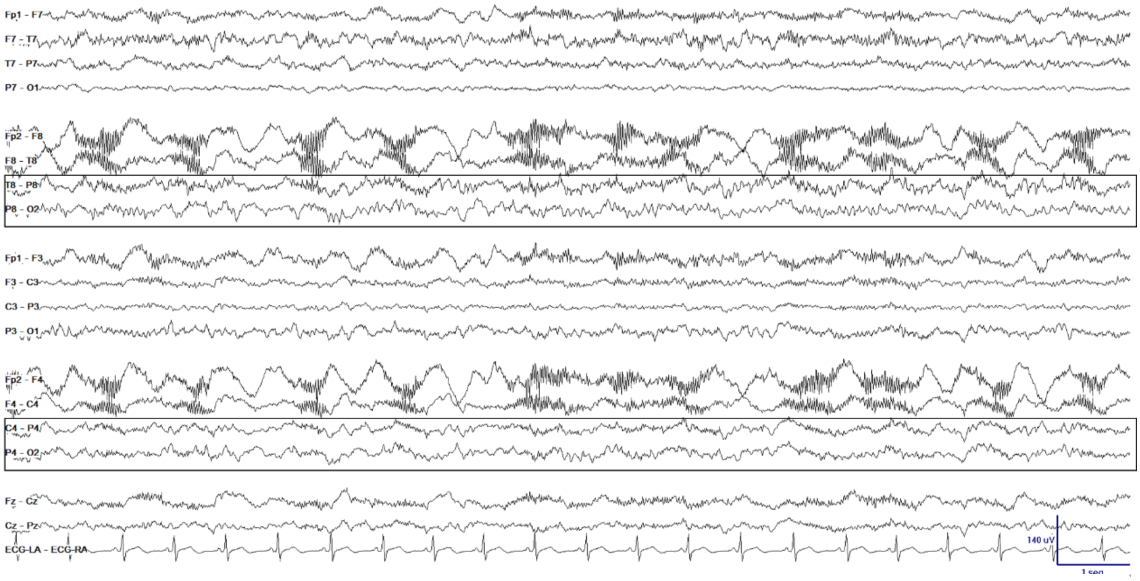

Fp1 - F7
F7 - T7
T7 - P7
P7 - O1
Fp2 - F8
F8 - T8
T8 - P8
P8 - O2
Fp1 - F3
F3 - C3
C3 - P3
P3 - O1
Fp2 - F4
F4 - C4
C4 - P4
P4 - O2
Fz - Cz
Cz - Pz
ECG-LA - ECG-RA
140 uV
1 sec

C 5

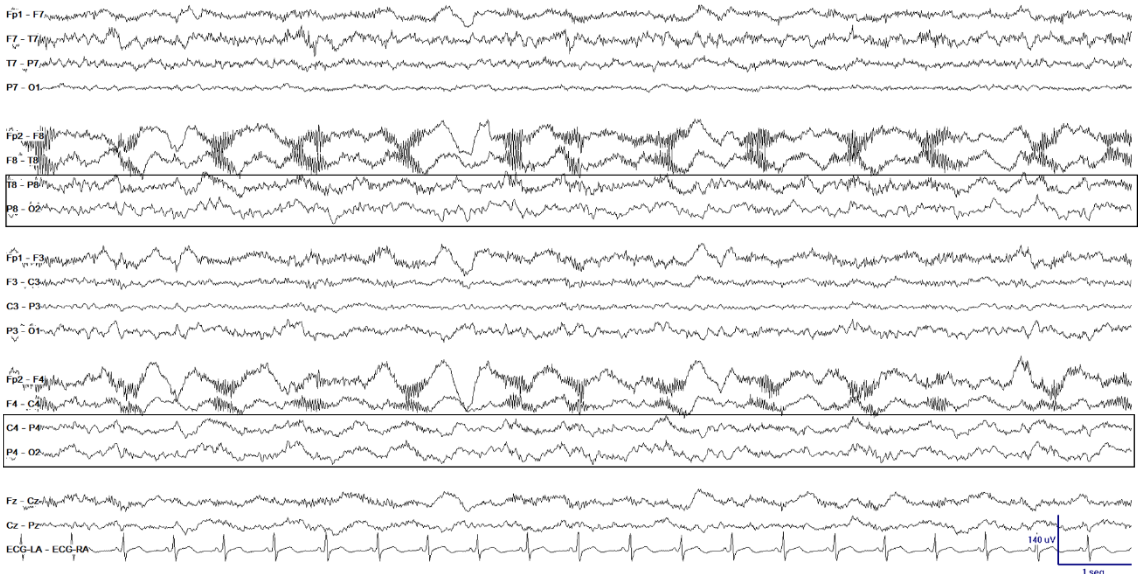

Fp1 - F7
F7 - T7
T7 - P7
P7 - O1
Fp2 - F8
F8 - T8
T8 - P8
P8 - O2
Fp1 - F3
F3 - C3
C3 - P3
P3 - O1
Fp2 - F4
F4 - C4
C4 - P4
P4 - O2
Fz - Cz
Cz - Pz
ECG-LA - ECG-RA
140 uV
1 sec

C 6

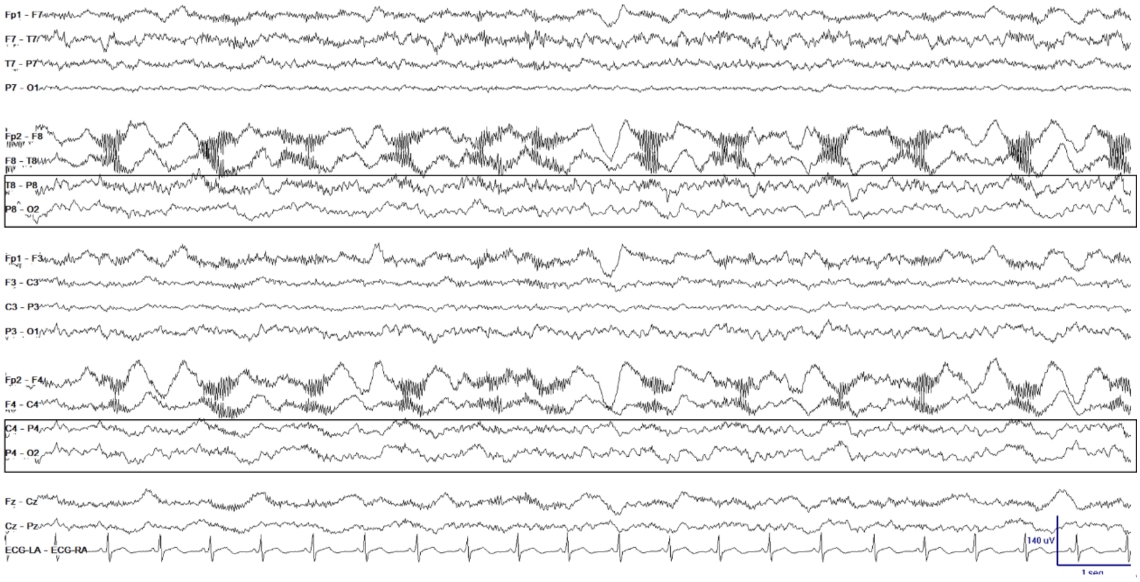
Fp1 - F7
F7 - T7
T7 - P7
P7 - O1
Fp2 - F8
F8 - T8
T8 - P8
P8 - O2
Fp1 - F3
F3 - C3
C3 - P3
P3 - O1
Fp2 - F4
F4 - C4
C4 - P4
P4 - O2
Fz - Cz
Cz - Pz
ECG-LA - ECG-RA
140 uV

C 7

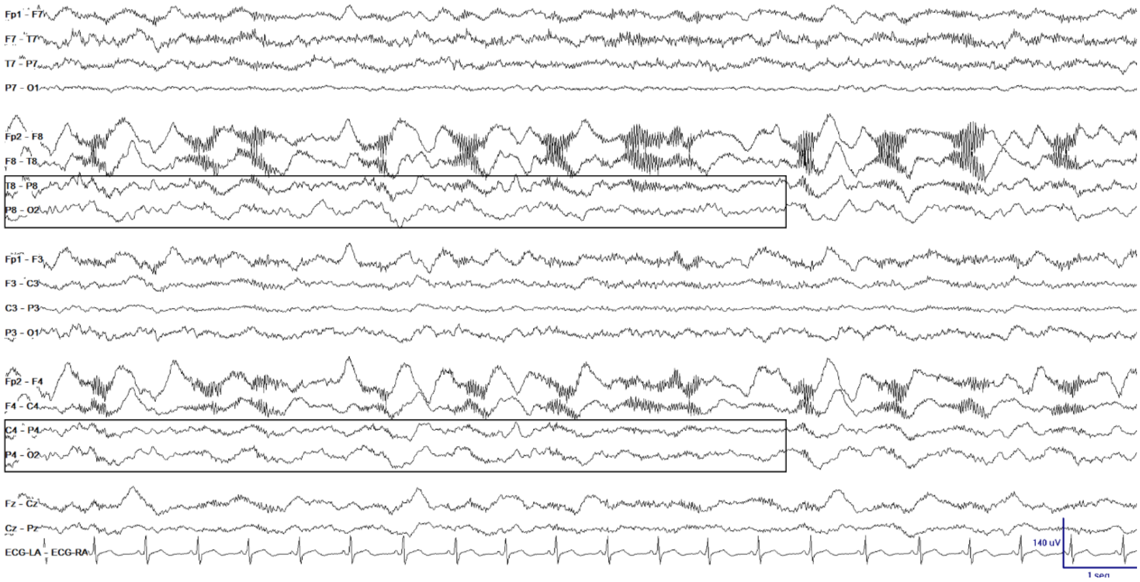
Fp1 - F7
F7 - T7
T7 - P7
P7 - O1
Fp2 - F8
F8 - T8
T8 - P8
P8 - O2
Fp1 - F3
F3 - C3
C3 - P3
P3 - O1
Fp2 - F4
F4 - C4
C4 - P4
P4 - O2
Fz - Cz
Cz - Pz
ECG-LA - ECG-RA
140 uV

C 8

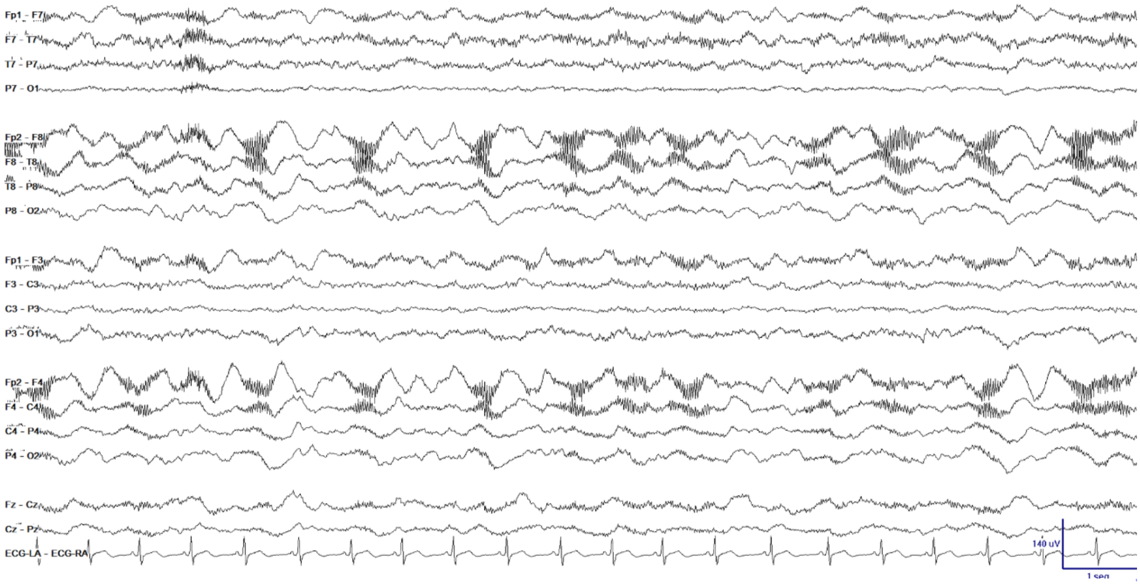
Fp1 - F7
F7 - T7
T7 - P7
P7 - O1
Fp2 - F8
F8 - T8
T8 - P8
P8 - O2
Fp1 - F3
F3 - C3
C3 - P3
P3 - O1
Fp2 - F4
F4 - C4
C4 - P4
P4 - O2
Fz - Cz
Cz - Pz
ECG-LA - ECG-RA
140 uV
1 sec

C 9

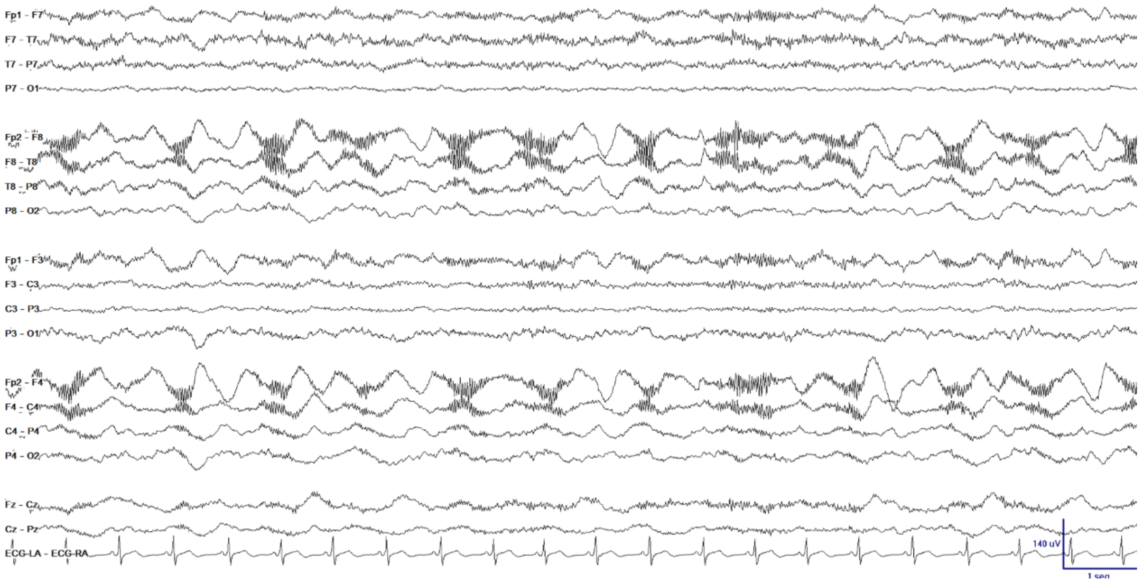
Fp1 - F7
F7 - T7
T7 - P7
P7 - O1
Fp2 - F8
F8 - T8
T8 - P8
P8 - O2
Fp1 - F3
F3 - C3
C3 - P3
P3 - O1
Fp2 - F4
F4 - C4
C4 - P4
P4 - O2
Fz - Cz
Cz - Pz
ECG-LA - ECG-RA
140 uV
1 sec

Supplementary Figure S2

Supplementary Figure S2(a)

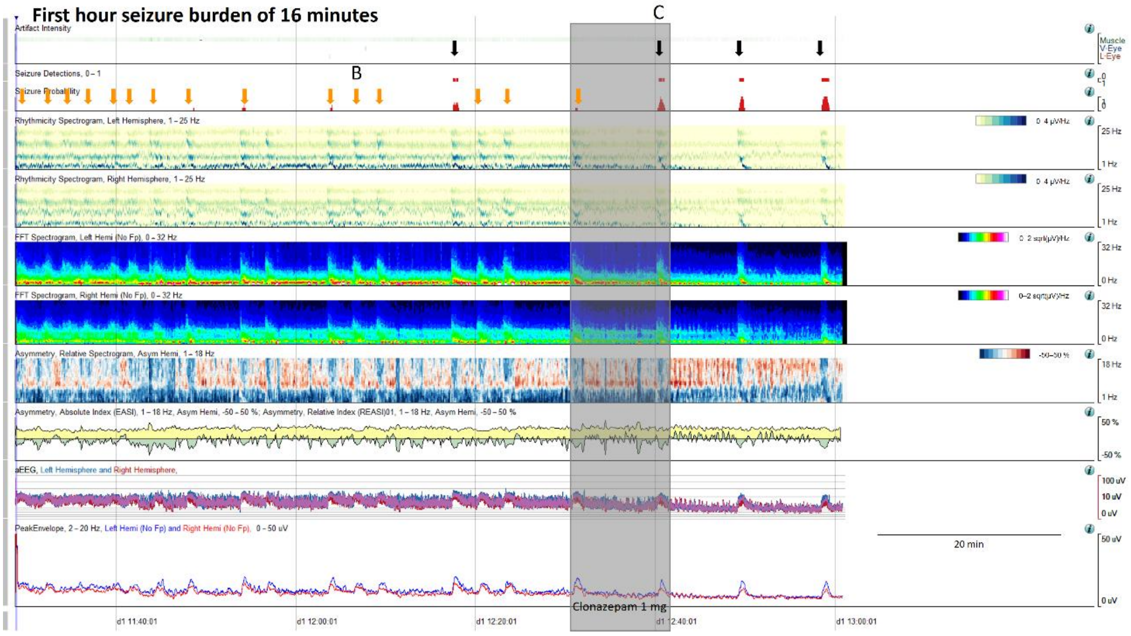

Figure S2(a). qEEG in a 2 hours display from a 73 -year-old man with past medial history of left ischemic stroke. In this panel is clearly visible, that seizure detector pointed 4 seizures (black arrows), however the seizure fingerprint is clearly recognizable in many more occasions (orange arrows) that are not detected by the algorithm, but clearly recognizable using all the qEEG trends, cheeking the raw EEG all the arrows turn out to be seizures (raw EEG at (B) on supplementary Figure S2(a)) and matching clinically with eye version to the right and nystagmus . Rhythmicity spectrogram show a clear increase in high frequency bands at the beginning of the seizure that move to slow frequency bands, always more pronounced in left hemisphere. Spectrogram depicted a solid flame in the left hemisphere a little bit recognizable also in the right hemisphere. Asymmetry spectrogram is quite interesting; all the time shows a great power in low frequencies in the left hemisphere, on the raw EEG correlates with delta waves in that hemisphere, and during the seizures also an increase of left power in high frequency bands. aEEG and peak envelope depicted the typical arch shape in each seizure that tell us about and increase in amplitude during the seizures. In supplementary Figure S2(a), you can find the raw EEG for the seizure pointed at (C). Note that after the antiseizure medication administration the seizures are more distant and became more well defined in evolution (a quite common situation in critical patients) therefore, easily recognizable by the automated seizure detector. First hour seizure burden was 16 minutes fulfilling the criteria of status epilepticus.

Raw EEG at (B1) shows an asymmetrical background. Right hemisphere with the presence of posterior alpha rhythm, and left hemisphere in delta range. There is also presence of some bilateral synchronous burst of delta waves. A seizure starts on the left posterior areas (B3) with small amplitude fast activity that evolve in location, frequency and morphology (from B3 to B6) with a time-locked right eyes version and nystagmus.

A similar seizure is shown in (C1) to (C5).

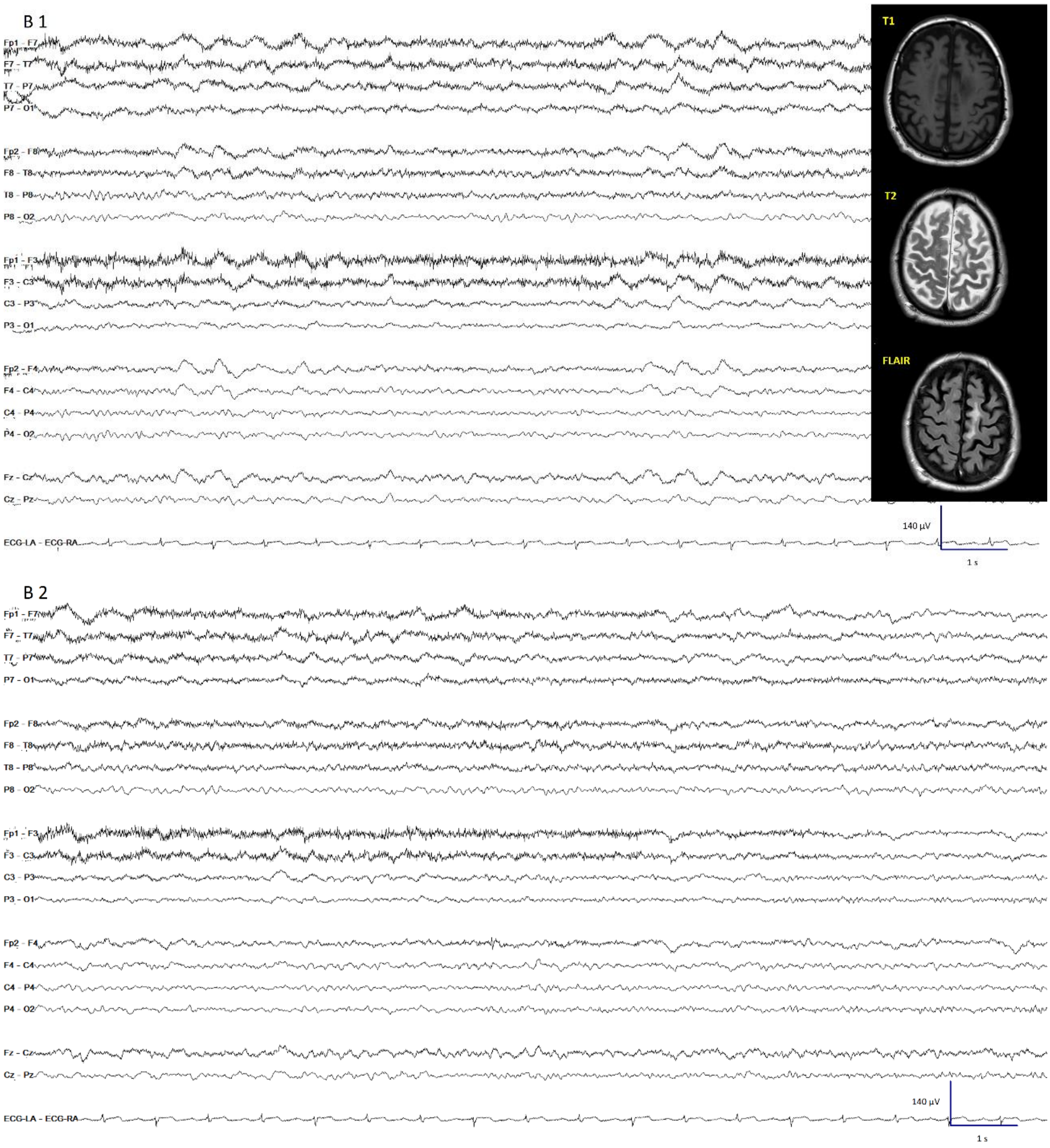
B 1
Fp1 - F7
F7 - T7
T7 - P7
P7 - O1
Fp2 - F8
F8 - T8
T8 - P8
P8 - O2
Fp1 - F3
F3 - C3
C3 - P3
P3 - O1
Fp2 - F4
F4 - C4
C4 - P4
P4 - O2
Fz - Cz
Cz - Pz
ECG-LA - ECG-RA
T1
T2
FLAIR
140 µV
1 s
B 2
Fp1 - F7
F7 - T7
T7 - P7
P7 - O1
Fp2 - F8
F8 - T8
T8 - P8
P8 - O2
Fp1 - F3
F3 - C3
C3 - P3
P3 - O1
Fp2 - F4
F4 - C4
C4 - P4
P4 - O2
Fz - Cz
Cz - Pz
ECG-LA - ECG-RA
140 µV
1 s

B 3

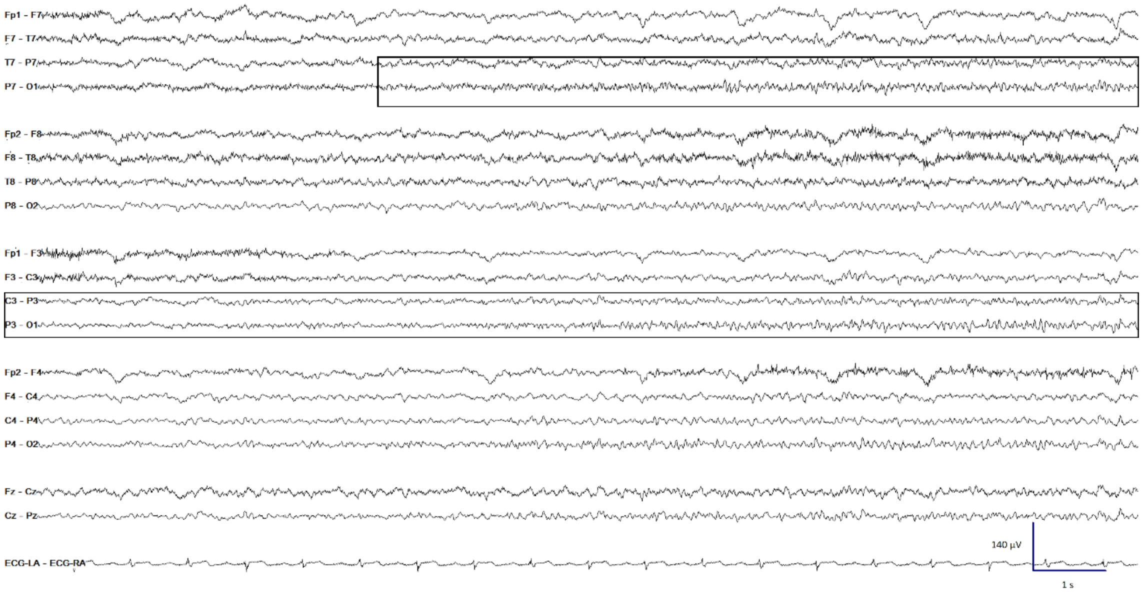

Fp1 - F7
F7 - T7
T7 - P7
P7 - O1
Fp2 - F8
F8 - T8
T8 - P8
P8 - O2
Fp1 - F3
F3 - C3
C3 - P3
P3 - O1
Fp2 - F4
F4 - C4
C4 - P4
P4 - O2
Fz - Cz
Cz - Pz
ECG-LA - ECG-RA
140 µV
1 s

B 4

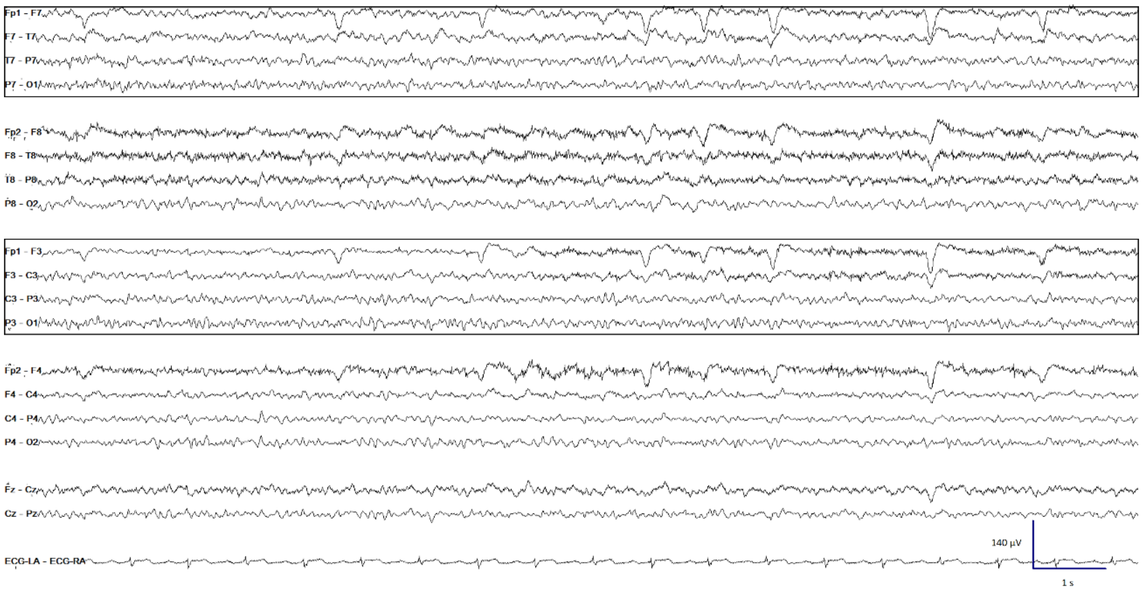

Fp1 - F7
F7 - T7
T7 - P7
P7 - O1
Fp2 - F8
F8 - T8
T8 - P8
P8 - O2
Fp1 - F3
F3 - C3
C3 - P3
P3 - O1
Fp2 - F4
F4 - C4
C4 - P4
P4 - O2
Fz - Cz
Cz - Pz
ECG-LA - ECG-RA
140 µV
1 s

B 5

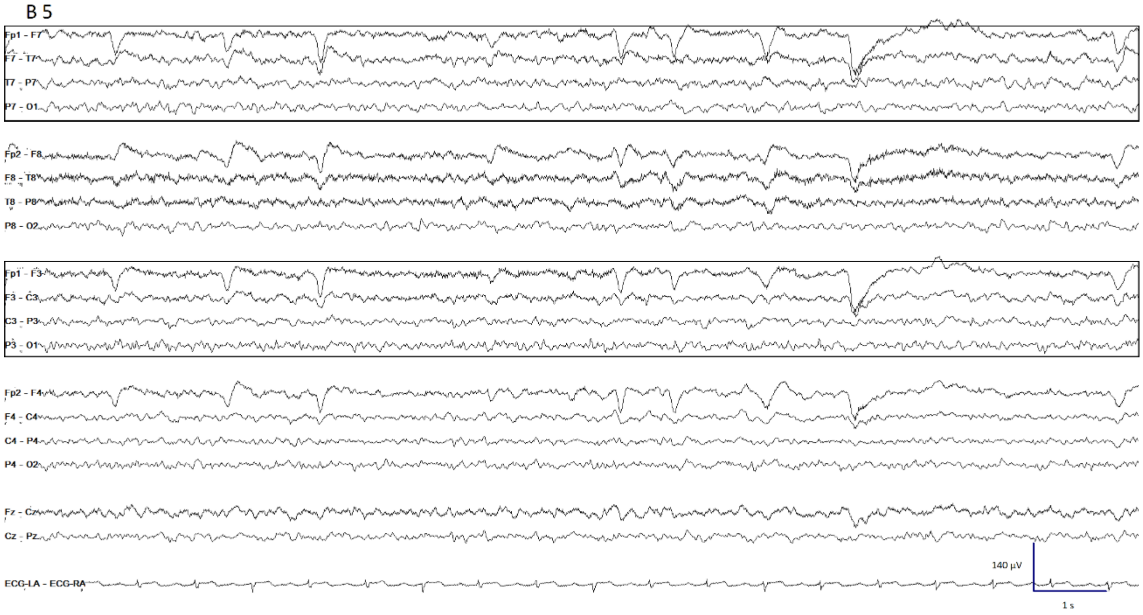

Fp1 - F7
F7 - T7
T7 - P7
P7 - O1
Fp2 - F8
F8 - T8
T8 - P8
P8 - O2
Fp1 - F3
F3 - C3
C3 - P3
P3 - O1
Fp2 - F4
F4 - C4
C4 - P4
P4 - O2
Fz - Cz
Cz - Pz
ECG-LA - ECG-RA
140 µV
1 s

B 6

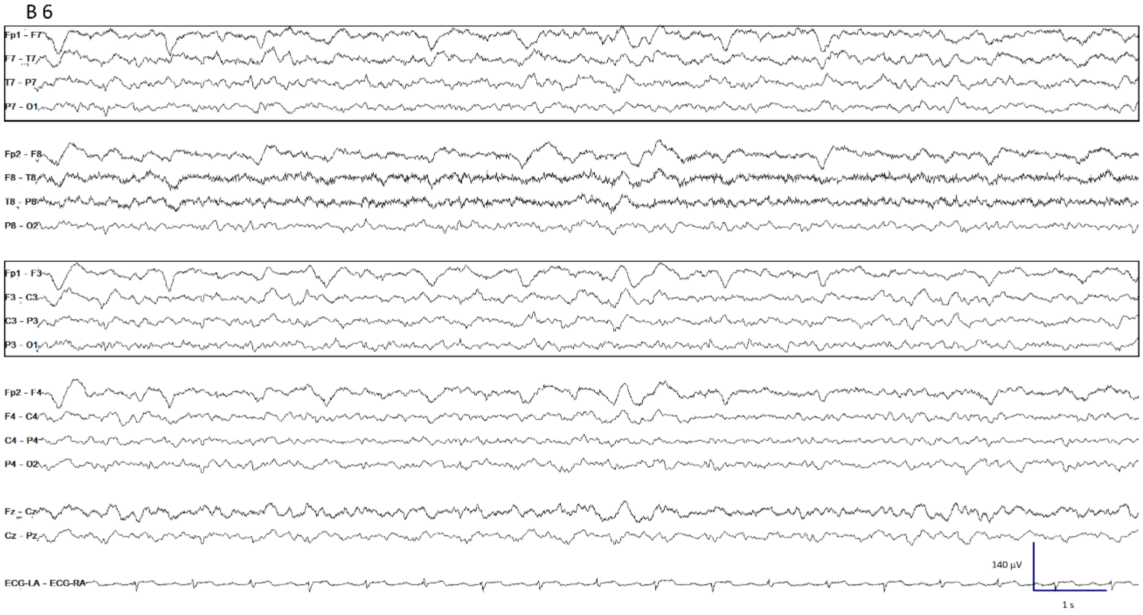

Fp1 - F7
F7 - T7
T7 - P7
P7 - O1
Fp2 - F8
F8 - T8
T8 - P8
P8 - O2
Fp1 - F3
F3 - C3
C3 - P3
P3 - O1
Fp2 - F4
F4 - C4
C4 - P4
P4 - O2
Fz - Cz
Cz - Pz
ECG-LA - ECG-RA
140 µV
1 s

B 7

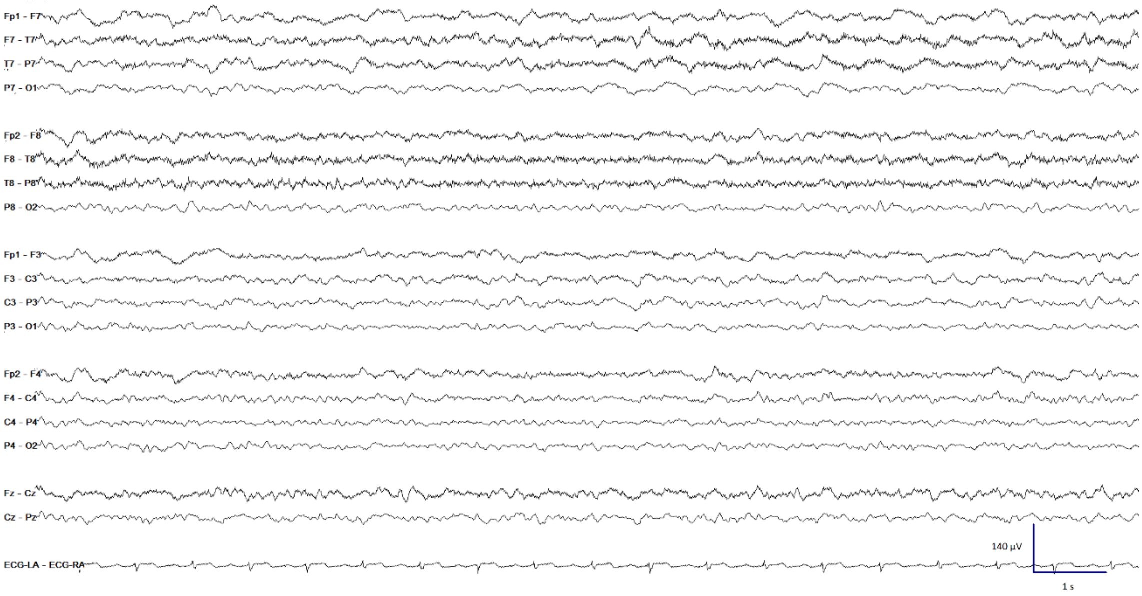
Fp1 - F7
F7 - T7
T7 - P7
P7 - O1
Fp2 - F8
F8 - T8
T8 - P8
P8 - O2
Fp1 - F3
F3 - C3
C3 - P3
P3 - O1
Fp2 - F4
F4 - C4
C4 - P4
P4 - O2
Fz - Cz
Cz - Pz
ECG-LA - ECG-RA
140 µV
1 s

C 1

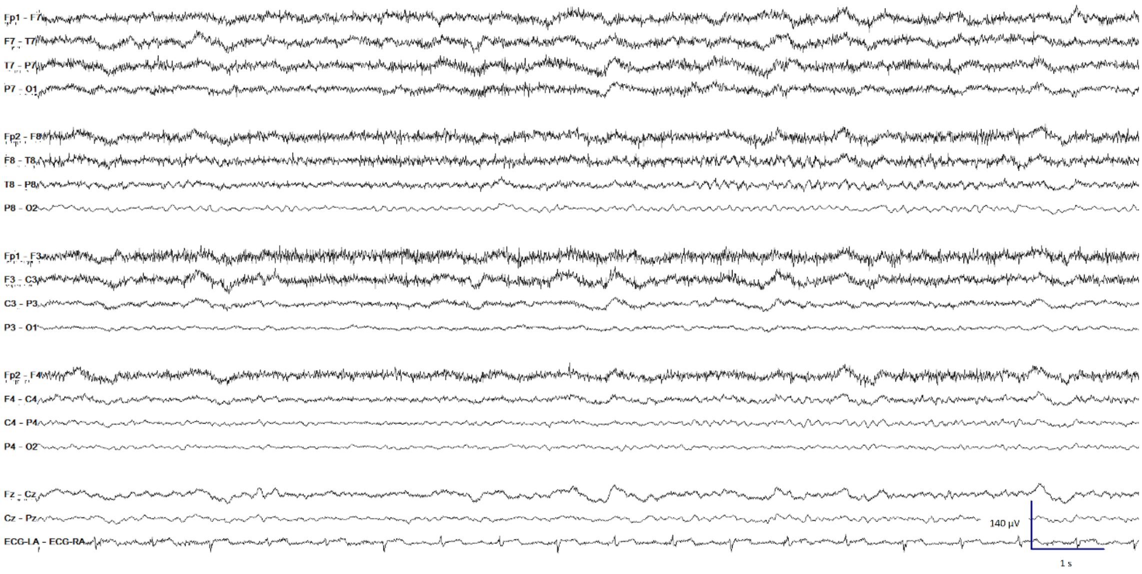
Fp1 - F7
F7 - T7
T7 - P7
P7 - O1
Fp2 - F8
F8 - T8
T8 - P8
P8 - O2
Fp1 - F3
F3 - C3
C3 - P3
P3 - O1
Fp2 - F4
F4 - C4
C4 - P4
P4 - O2
Fz - Cz
Cz - Pz
ECG-LA - ECG-RA
140 µV
1 s

C 2

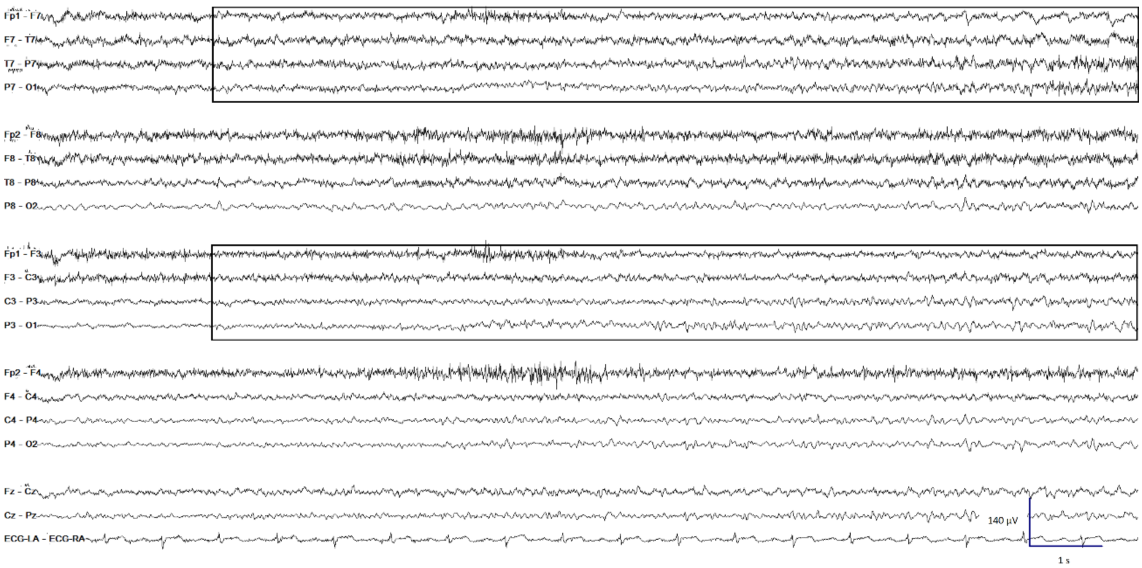
Fp1 - F7
F7 - T7
T7 - P7
P7 - O1
Fp2 - F8
F8 - T8
T8 - P8
P8 - O2
Fp1 - F3
F3 - C3
C3 - P3
P3 - O1
Fp2 - F4
F4 - C4
C4 - P4
P4 - O2
Fz - Cz
Cz - Pz
ECG-LA - ECG-RA
140 µV
1 s

C 3

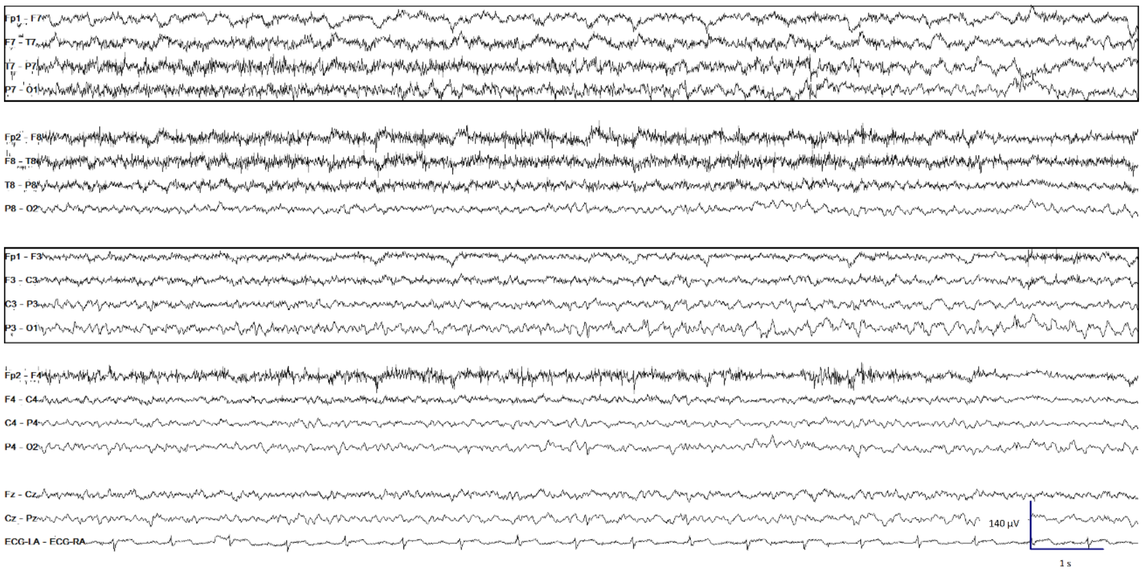
Fp1 - F7
F7 - T7
T7 - P7
P7 - O1
Fp2 - F8
F8 - T8
T8 - P8
P8 - O2
Fp1 - F3
F3 - C3
C3 - P3
P3 - O1
Fp2 - F4
F4 - C4
C4 - P4
P4 - O2
Fz - Cz
Cz - Pz
ECG-LA - ECG-RA
140 µV
1 s

C 4

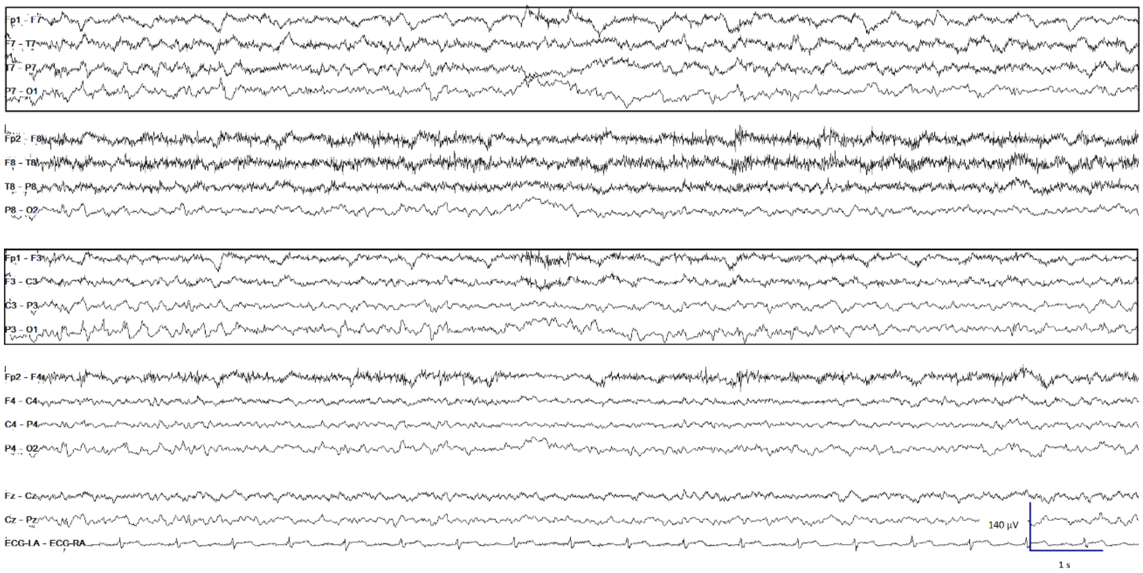
Fp1 - F7
F7 - T7
T7 - P7
P7 - O1
Fp2 - F8
F8 - T8
T8 - P8
P8 - O2
Fp1 - F3
F3 - C3
C3 - P3
P3 - O1
Fp2 - F4
F4 - C4
C4 - P4
P4 - O2
Fz - Cz
Cz - Pz
ECG-LA - ECG-RA
140 µV
1 s

C 5

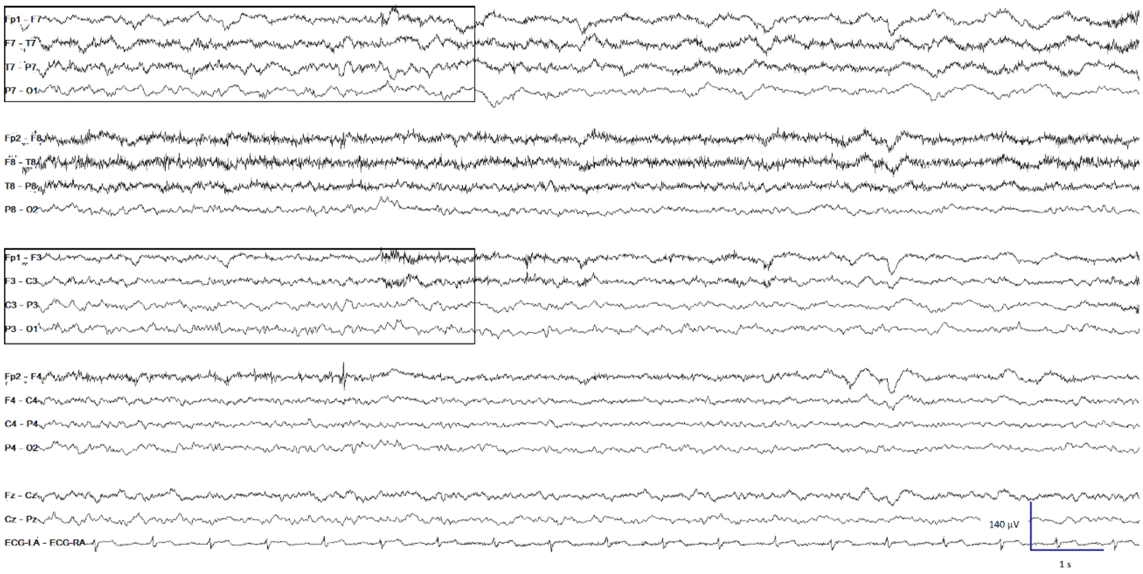
Fp1 - F7
F7 - T7
T7 - P7
P7 - O1
Fp2 - F8
F8 - T8
T8 - P8
P8 - O2
Fp1 - F3
F3 - C3
C3 - P3
P3 - O1
Fp2 - F4
F4 - C4
C4 - P4
P4 - O2
Fz - Cz
Cz - Pz
ECG-LA - ECG-RA
140 µV
1 s

Supplementary figure S2(c)

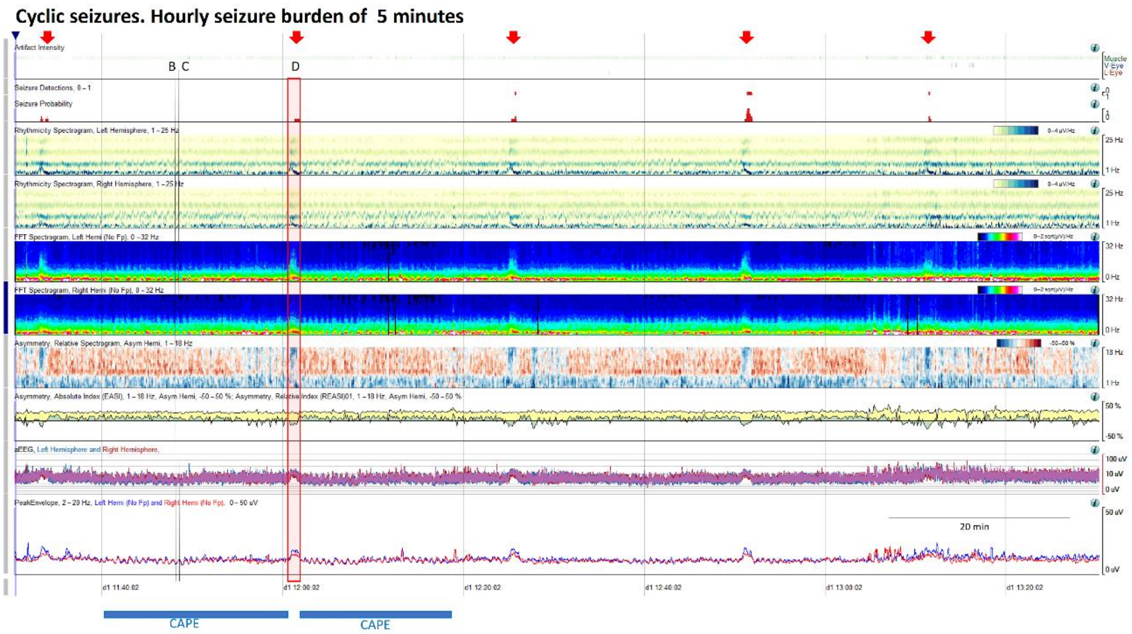

Supplementary Figure S2(c). Cyclic alternating pattern of encephalopathy (CAPE) intermingled with seizures (red arrows). A cyclic alternating pattern is visible in qEEG (blue lines) alternating two EEG patterns in a regular manner, one of them (raw EEG at (B) exhibits theta frequencies of normal amplitude, and also intermingled with delta irregular waves on the left temporal,while the other pattern shows more slowing and slightly higher amplitudes (raw EEG at C). Note the difference between cyclic alternating pattern more arch shape on the spectrogram (blue line) and seizures (red arrows) which have more triangular shape and smooth edges. Correlations with the raw EEG at (D) are shown in the supplementary figure S2(c) (D1) to (D7).

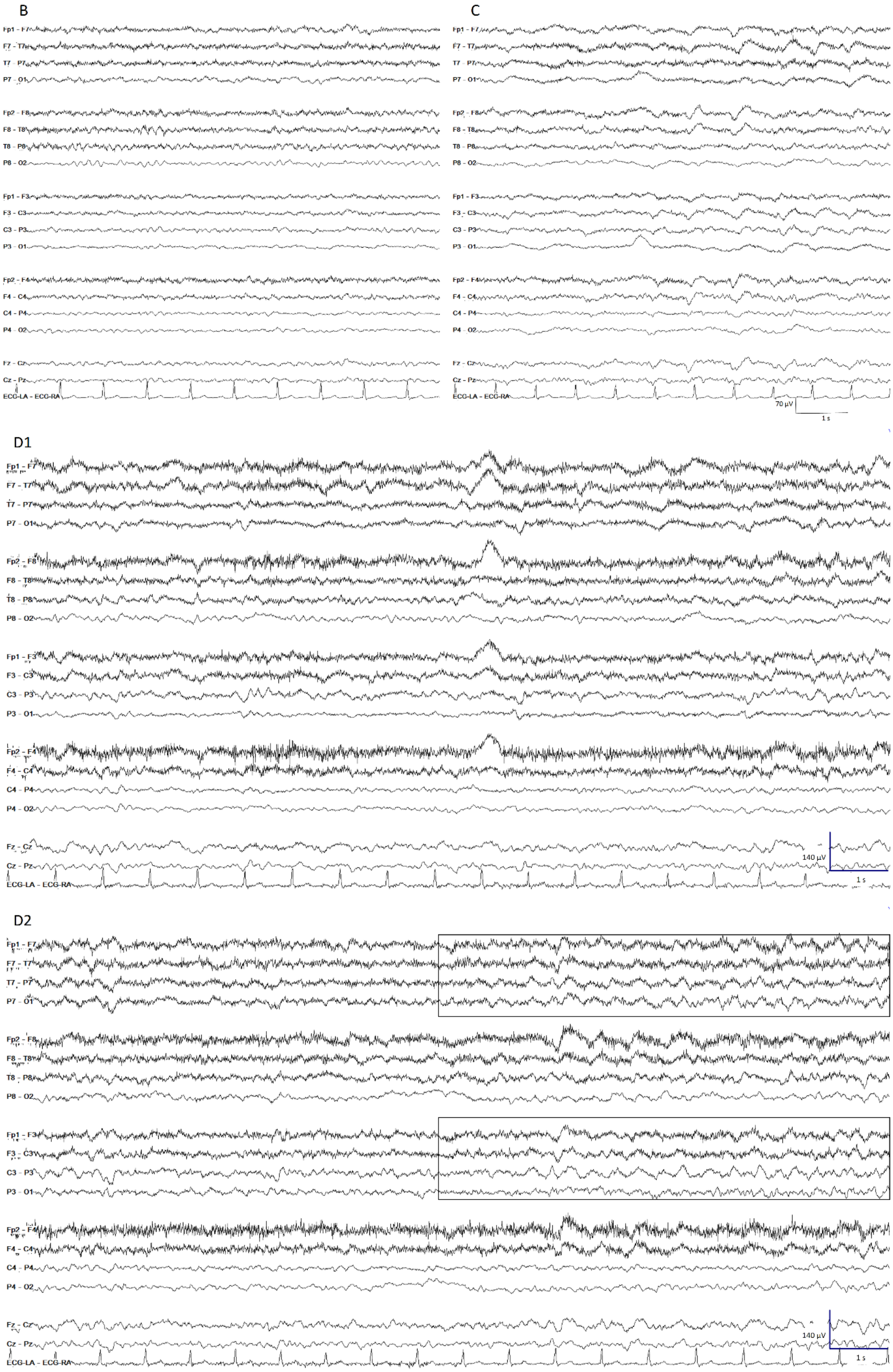
B
C
70 µV
1 s
D1
140 µV
1 s
D2
140 µV
1 s

D3

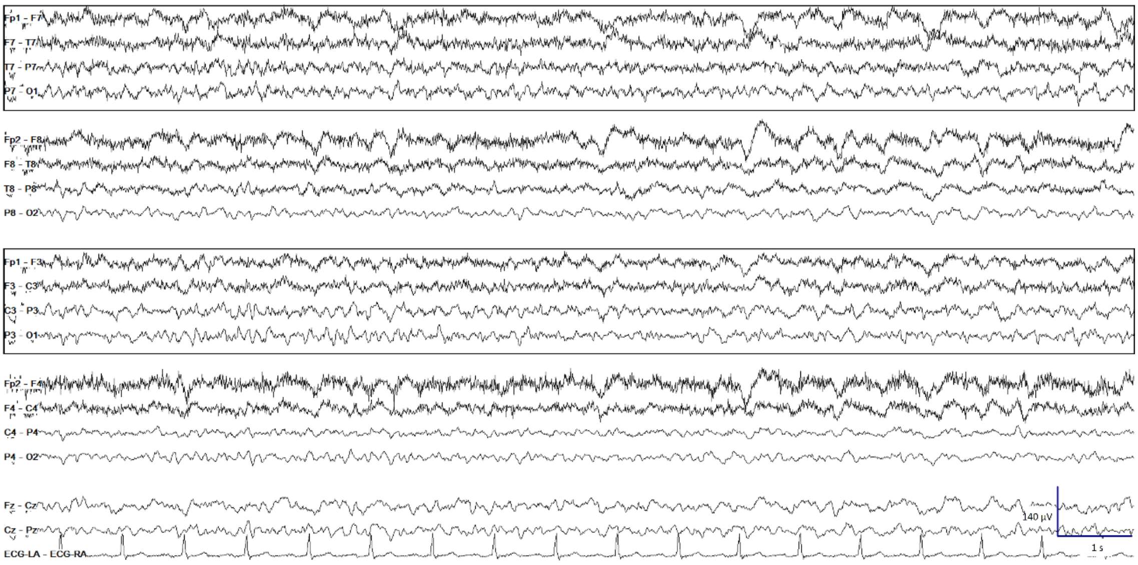

Fp1 - F7
F7 - T7
T7 - P7
P7 - O1
Fp2 - F8
F8 - T8
T8 - P8
P8 - O2
Fp1 - F3
F3 - C3
C3 - P3
P3 - O1
Fp2 - F4
F4 - C4
C4 - P4
P4 - O2
Fz - Cz
Cz - Pz
ECG-LA - ECG-RA
140 µV
1 s

D4

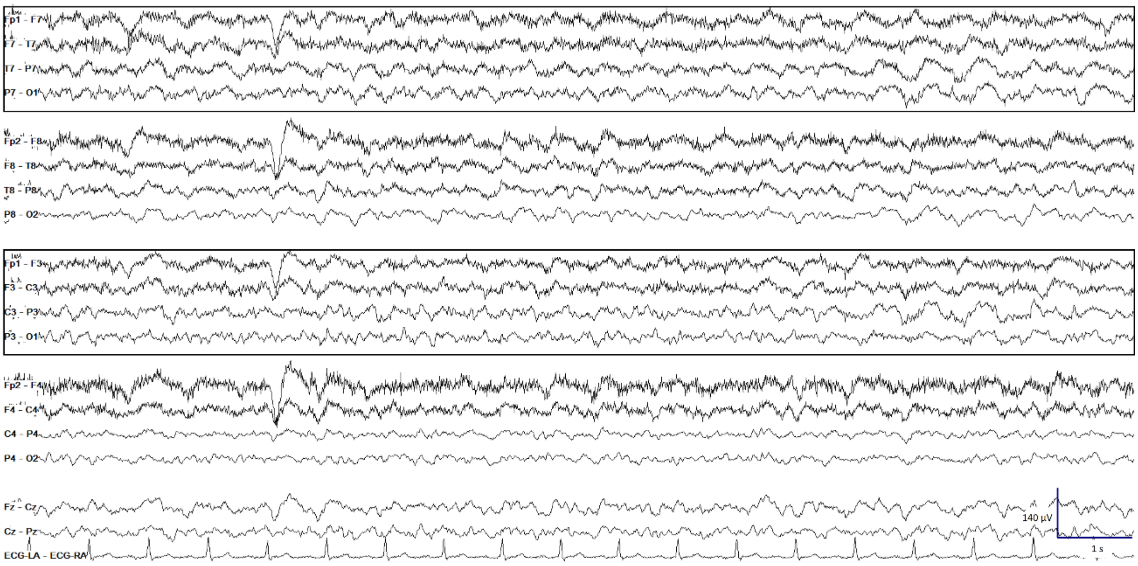

Fp1 - F7
F7 - T7
T7 - P7
P7 - O1
Fp2 - F8
F8 - T8
T8 - P8
P8 - O2
Fp1 - F3
F3 - C3
C3 - P3
P3 - O1
Fp2 - F4
F4 - C4
C4 - P4
P4 - O2
Fz - Cz
Cz - Pz
ECG-LA - ECG-RA
140 µV
1 s

D5

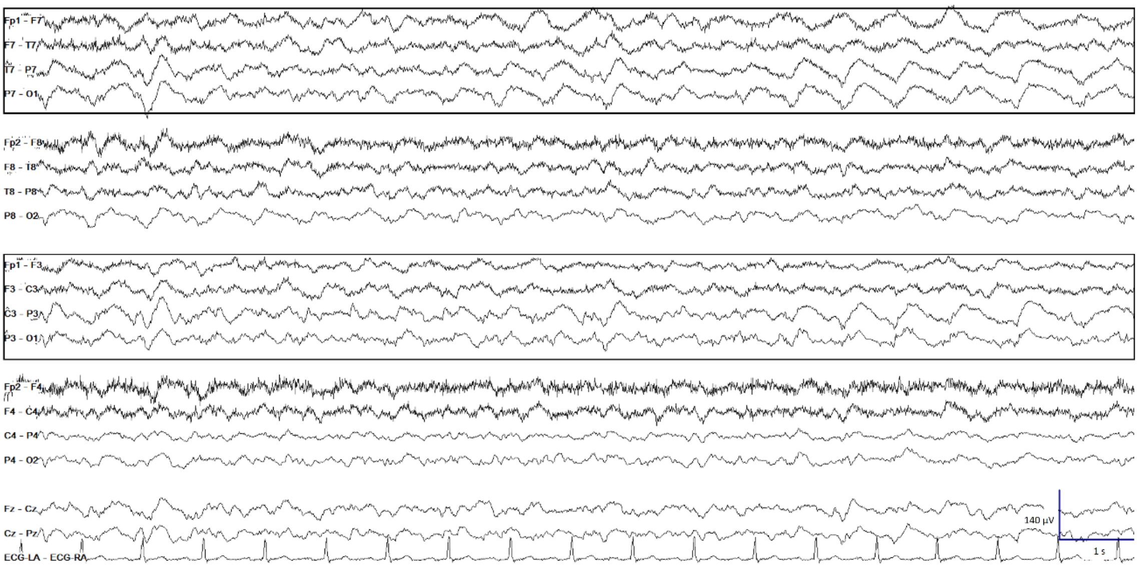

Fp1 - F7
F7 - T7
T7 - P7
P7 - O1
Fp2 - F8
F8 - T8
T8 - P8
P8 - O2
Fp1 - F3
F3 - C3
C3 - P3
P3 - O1
Fp2 - F4
F4 - C4
C4 - P4
P4 - O2
Fz - Cz
Cz - Pz
ECG-LA - ECG-RA
140 µV
1 s

D6

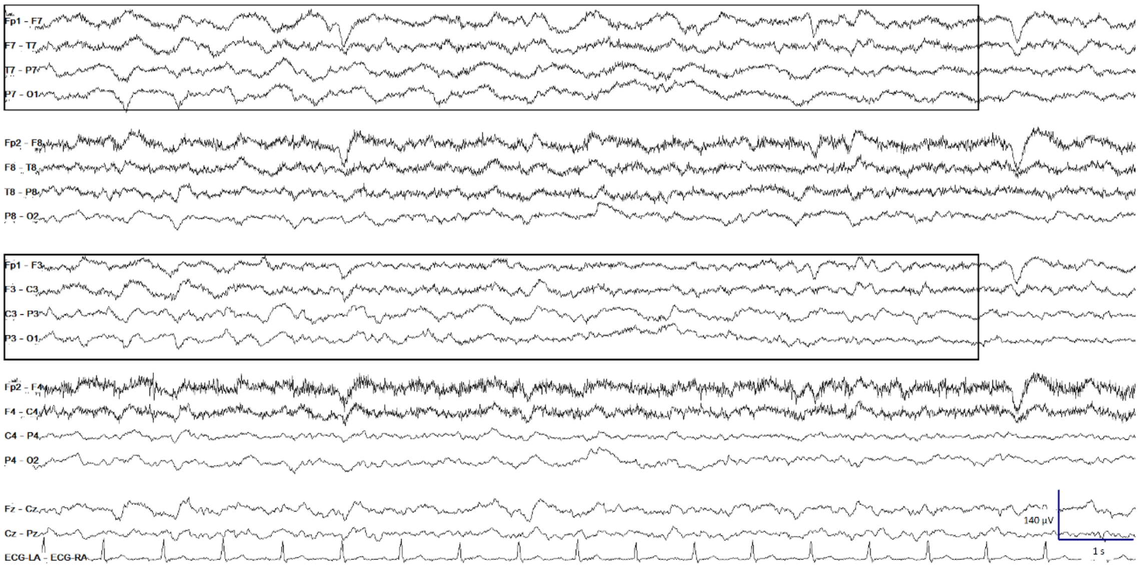
Fp1 - F7
F7 - T7
T7 - P7
P7 - O1
Fp2 - F8
F8 - T8
T8 - P8
P8 - O2
Fp1 - F3
F3 - C3
C3 - P3
P3 - O1
Fp2 - F4
F4 - C4
C4 - P4
P4 - O2
Fz - Cz
Cz - Pz
ECG-LA - ECG-RA
140 µV
1 s

D7

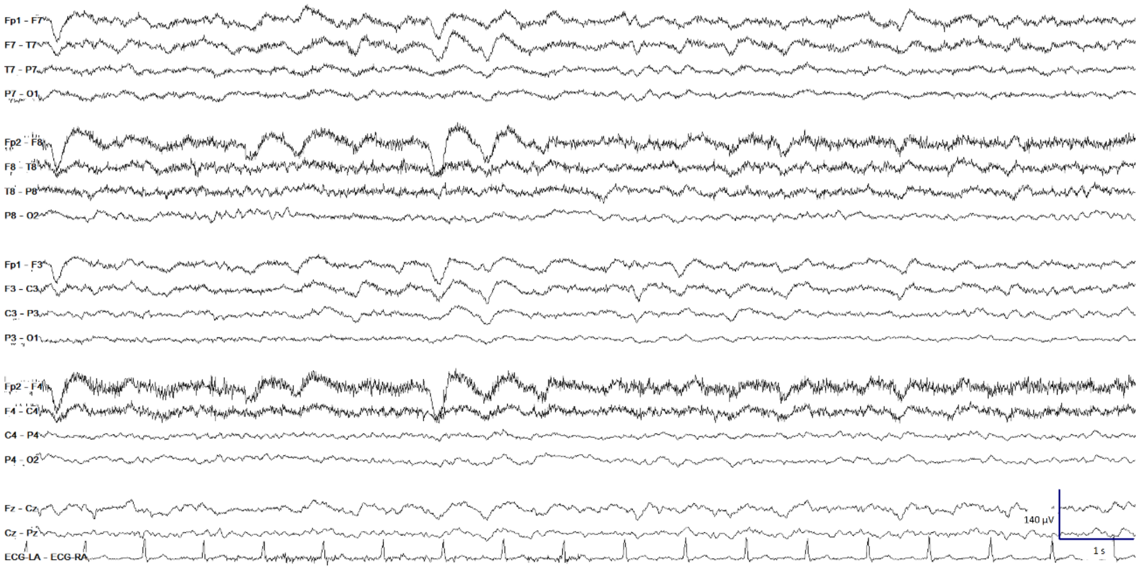
Fp1 - F7
F7 - T7
T7 - P7
P7 - O1
Fp2 - F8
F8 - T8
T8 - P8
P8 - O2
Fp1 - F3
F3 - C3
C3 - P3
P3 - O1
F4 - C4
C4 - P4
P4 - O2
Fz - Cz
Cz - Pz
ECG-LA - ECG-RA
140 µV
1 s

Supplementary Figure S3

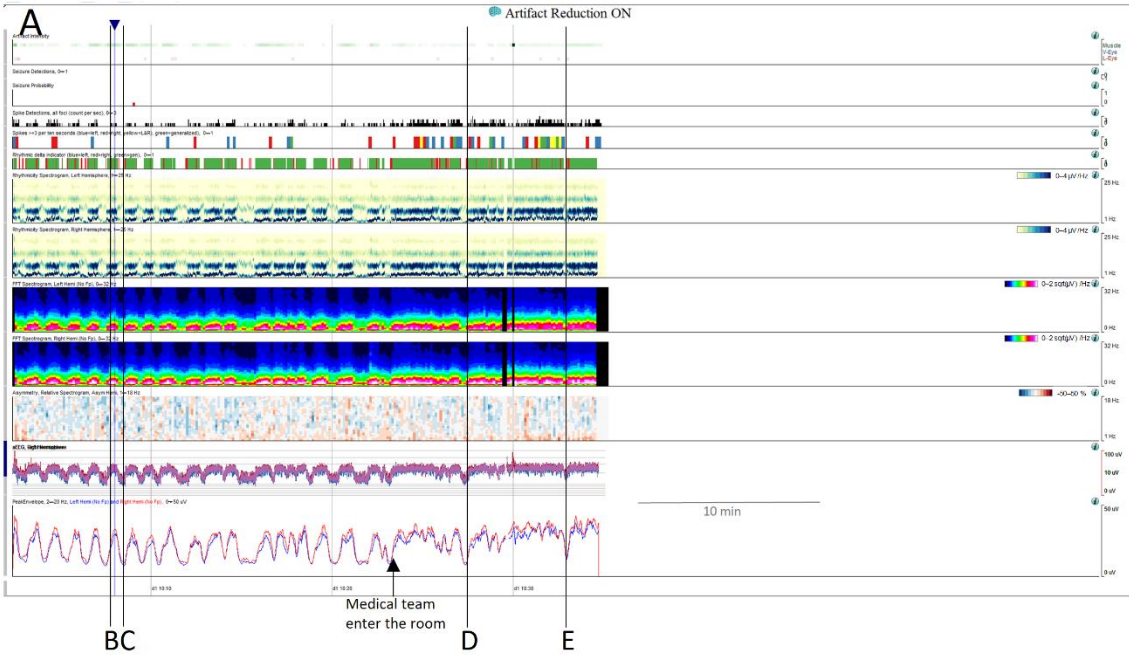

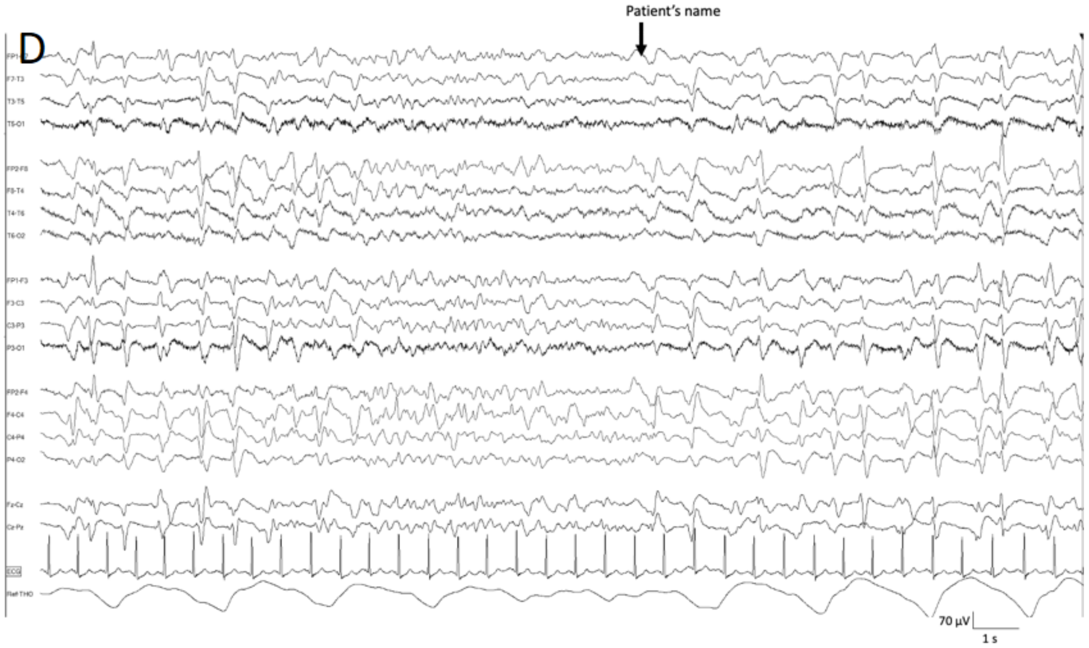

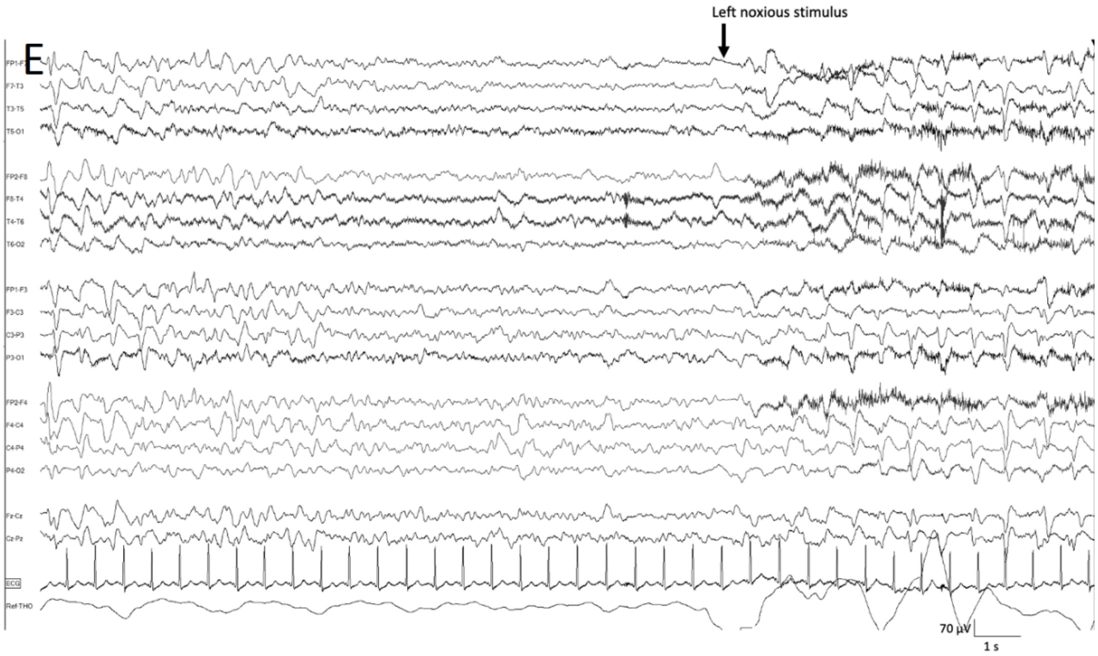

Supplementary Figure S3: Cyclic alternating pattern of encephalopathy (CAPE): (D) acoustic stimulation (patient's name) and noxious stimulation (E) induced the GPD pattern. GPDs pattern fulfils the criteria of IIC and in this specific patient, as it happens in many encephalopathic patients, correspond to the most stimulated state, despite the theta pattern looks like, apparently, more normal.
